# Supplementary material for: Evolution of DNA replication origin specification and gene silencing mechanisms
Source: Nat Commun. 2020 Oct 14;11:5175. doi: 10.1038/s41467-020-18964-x (PMC7560902; doi:10.1038/s41467-020-18964-x)
Supplement: Supplementary file 1 — Supplementary Information [file 41467_2020_18964_MOESM1_ESM.pdf]

Evolution of DNA Replication Origin Specification and Gene Silencing Mechanisms

Hu et al.

Supplementary Information

Supplementary Figures 1-13

Supplementary Tables 1-3

Supplementary Methods Tables 1-2

Supplementary Figures

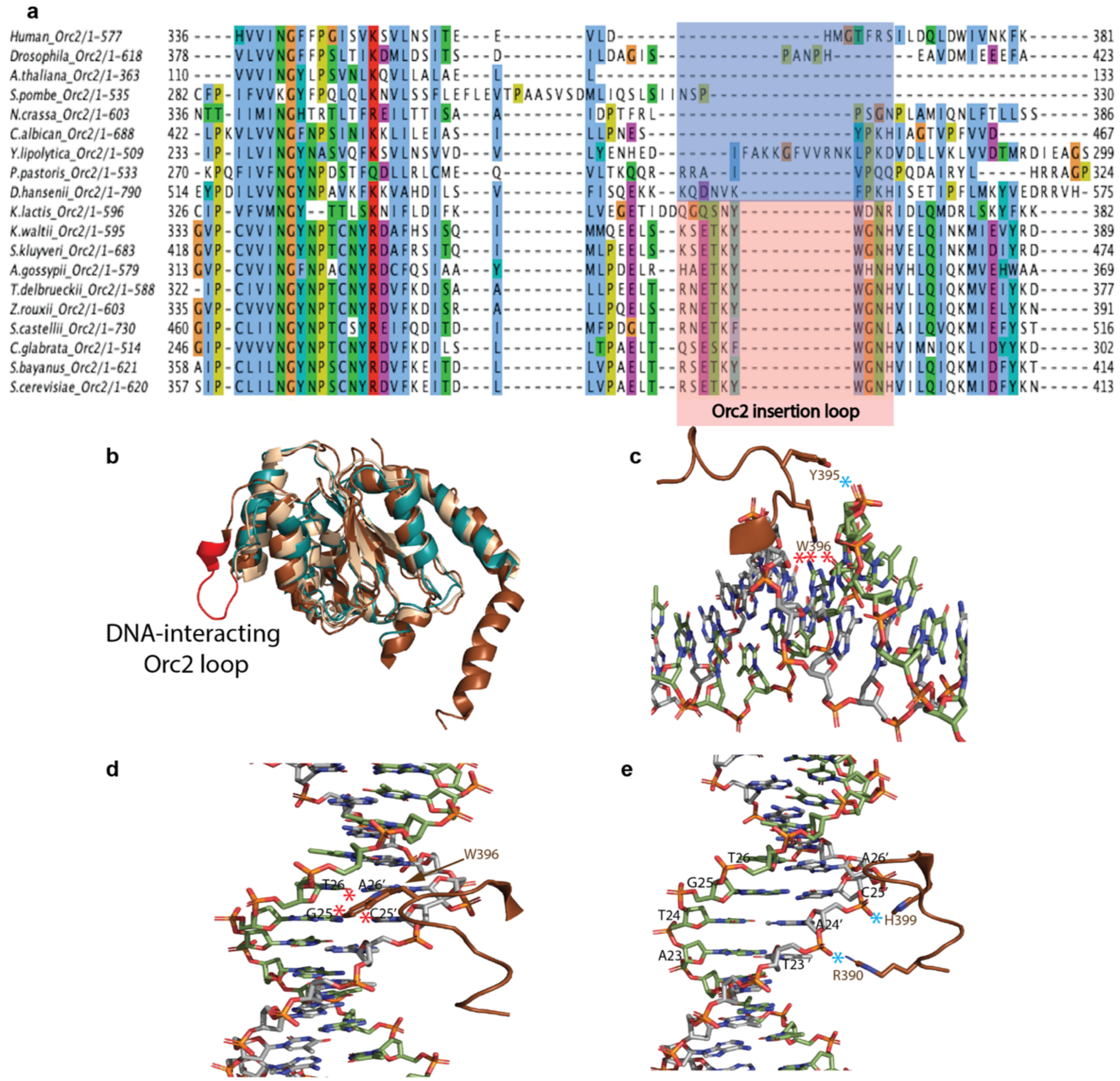

Supplementary Fig. 1 | Orc2 loop DNA interaction exists in species with origin sequence specificity. a, Multiple sequence alignment of Orc2 among representing eukaryotic species as indicated. Orc2 DNA interacting loop region indicated with species that don't have sequence specific origins shadowed in blue and species that sequence specific origins exist shadowed in pink. **b,** Orc2 AAA+ domain structure superposition among human Orc2 in teal color (from PDB code 5uj7), *Drosophila* Orc2 in wheat color (from PDB code 4xgc) and *S. cerevisiae* Orc2 in brown color (from PDB code 5udb). Orc2 loop that interact with DNA is colored in red. **c-e,** R390, Y395, W396, and H399 interact with DNA in base-specific (specificity) and base-nonspecific (affinity) manner in ORC-DNA structure at 3Å (PDB code 5zr1). Red asterisks denote the base interaction between amino acid and DNA base. Blue asterisks denote the base-nonspecific interaction between amino acid and DNA phosphate backbone. Prime symbols denote bases on

the opposite strand. Bases numbering denote as the positions in logo (see Fig. 2b). **c** shows Orc2 loop origin DNA minor groove insertion with base-specific interaction between W396 and G25, C25' and T26, and base-nonspecific interaction between Y395 and phosphate backbone of T27. **d-e,** same as in **c**, but view in different angles. **d** shows the base-specific interaction between W396 and G25, C25' and T26. **e** shows the base-nonspecific interaction between R390 and phosphate backbone of T23' and base-nonspecific interaction between H399 and phosphate backbone of A24'.

orc4Δ::TRP1 + p*ORC4/URA3* + plasmid indicated below:

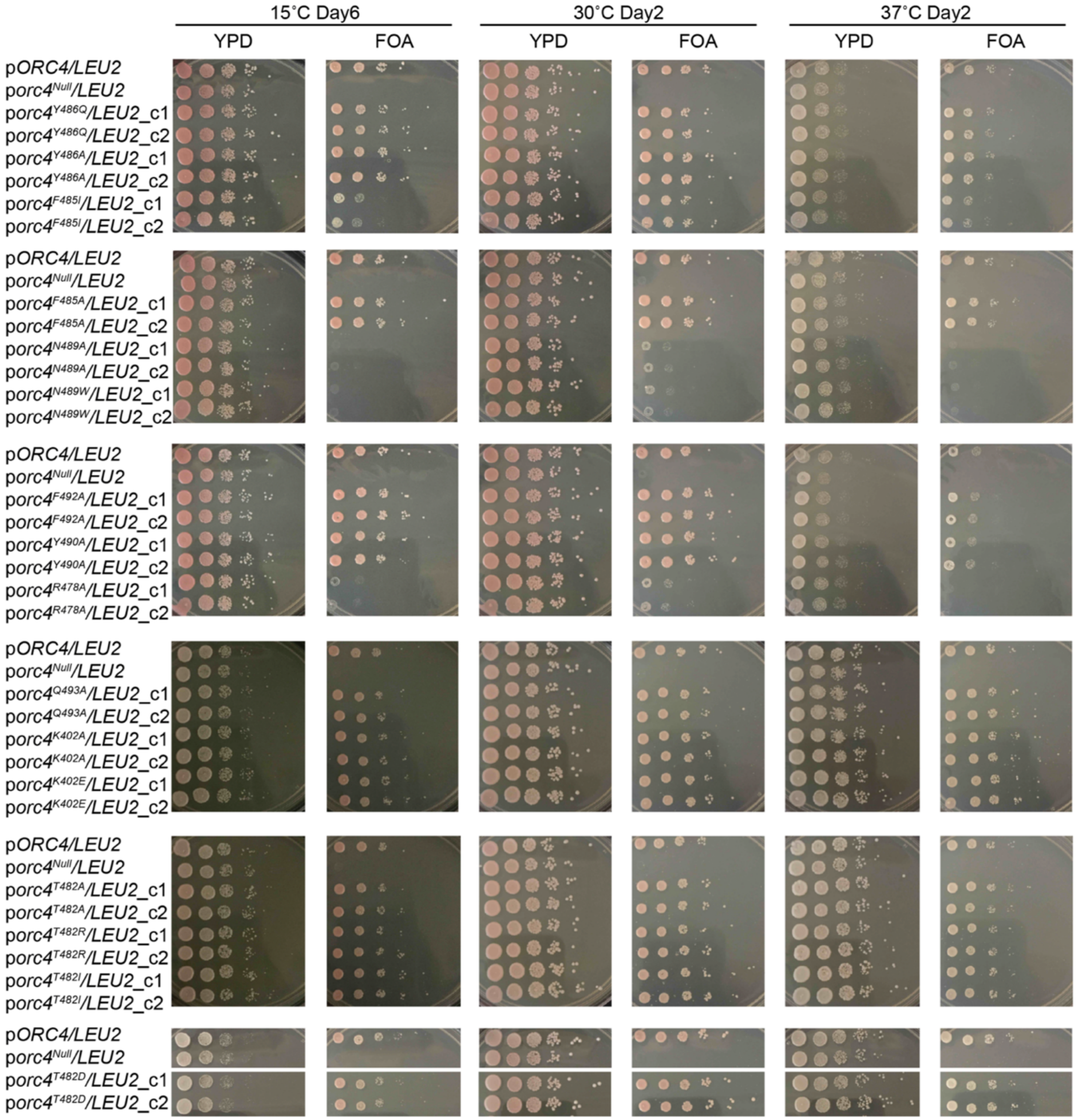

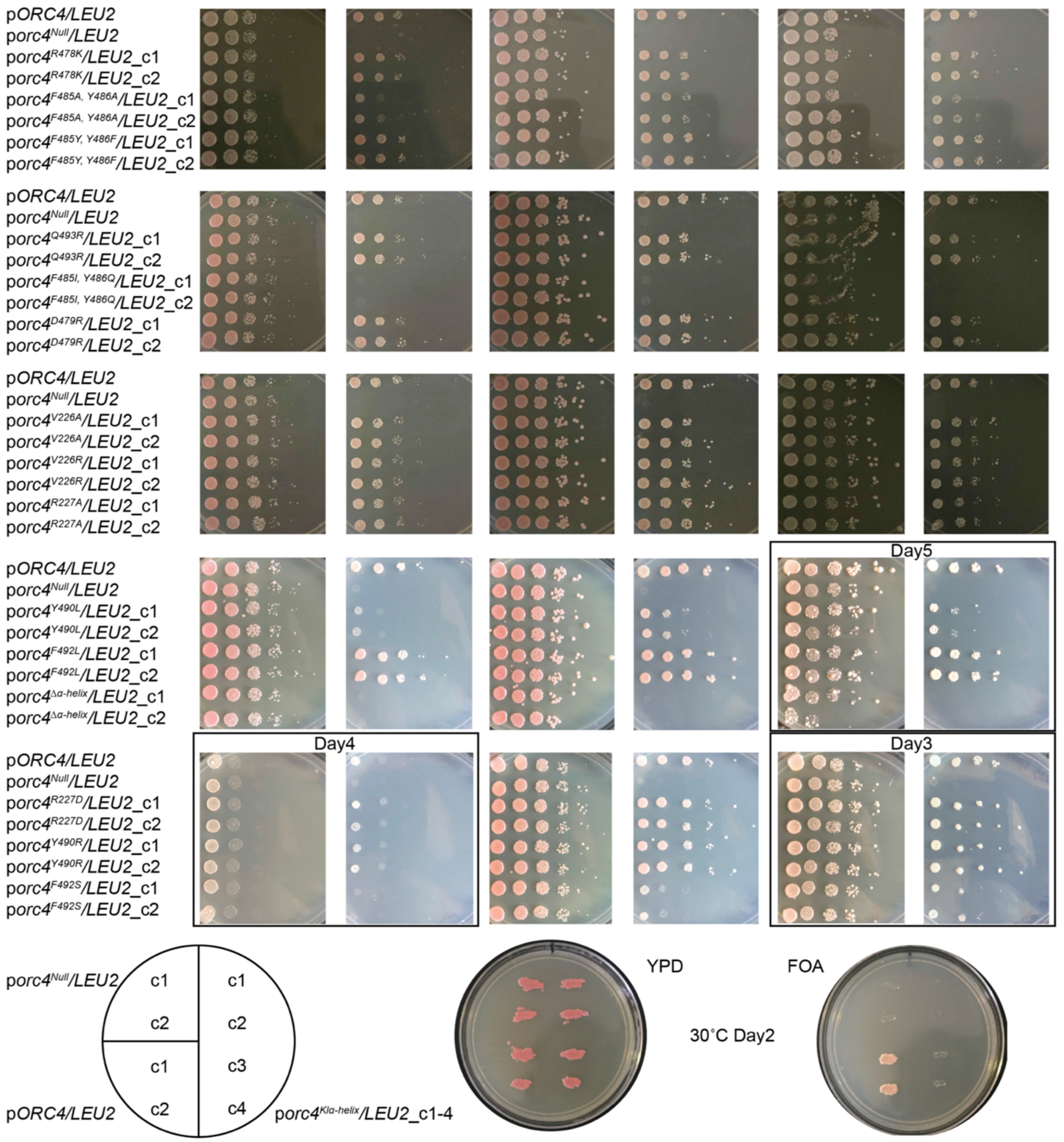

Supplementary Fig. 2 | Orc4 mutants viability phenotypes in plasmid shuffle assay. Detailed procedure of plasmid shuffle assay is described in Method, Plasmid shuffle assay. Briefly, strains (*orc4*Δ*::TRP1* + p*ORC4/URA3* + p*orc4*$^{tested\ allele}$/*LEU2*) were grown overnight in YPD and spotted onto 5-FOA plates with 10-fold serial dilutions starting from 1.5x10^7 cells and spotted onto YPD plates as control. Mutations were indicated. Strain (*orc4*Δ*::TRP1* + p*ORC4/URA3* + p*ORC4/LEU2*) and strain (*orc4*Δ*::TRP1* + p*ORC4/URA3* + p*orc4*^{null}/*LEU2*) were spotted as controls. Biological duplicates are denoted as c1 and c2. Plates were cultured under 25˚C, 30˚C, or 37˚C for different days (as indicated) to test their cold or temperature sensitivity. *Orc4* mutant phenotypes summarized in Supplementary Table 1.

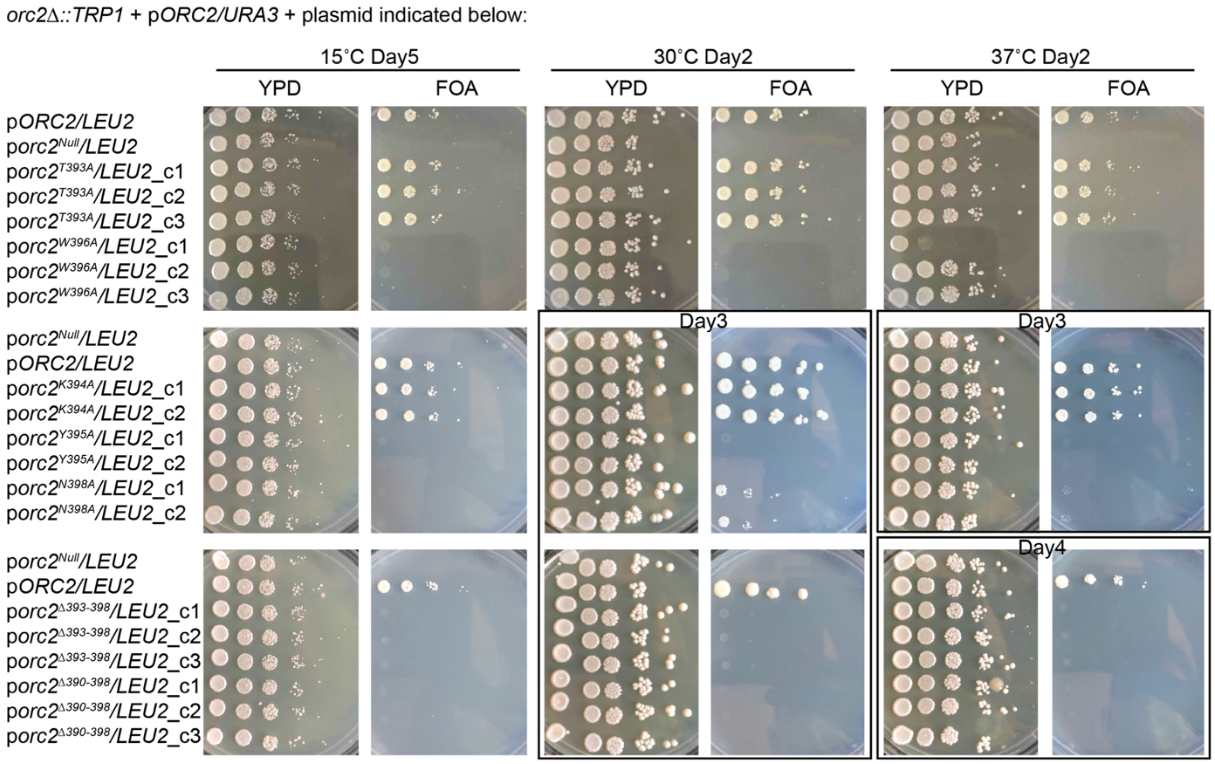

Supplementary Fig. 3 | *Orc2* mutants viability phenotypes in plasmid shuffle assay. Detailed procedure of plasmid shuffle assay is described in Method, Plasmid shuffle assay. Briefly, strains (*orc2*Δ*::TRP1* + p*ORC2/URA3* + p*orc2*$^{tested\ allele}$/*LEU2*) were grown overnight in YPD and spotted onto 5-FOA plates with 10-fold serial dilutions starting from 1.5x10^{7} cells and spotted onto YPD plates as control. Mutations were indicated. Strain (*orc2*Δ*::TRP1* + p*ORC2/URA3* + p*ORC2/LEU2*) and strain (*orc2*Δ*::TRP1* + p*ORC2/URA3* + p*orc2*^{null}/*LEU2*) were spotted as controls. Biological duplicates are denoted as c1 and c2. Plates were cultured under 25˚C, 30˚C, or 37˚C for different days (as indicated) to test their cold or temperature sensitivity. *Orc2* mutant phenotype summarized in Supplementary Table 2.

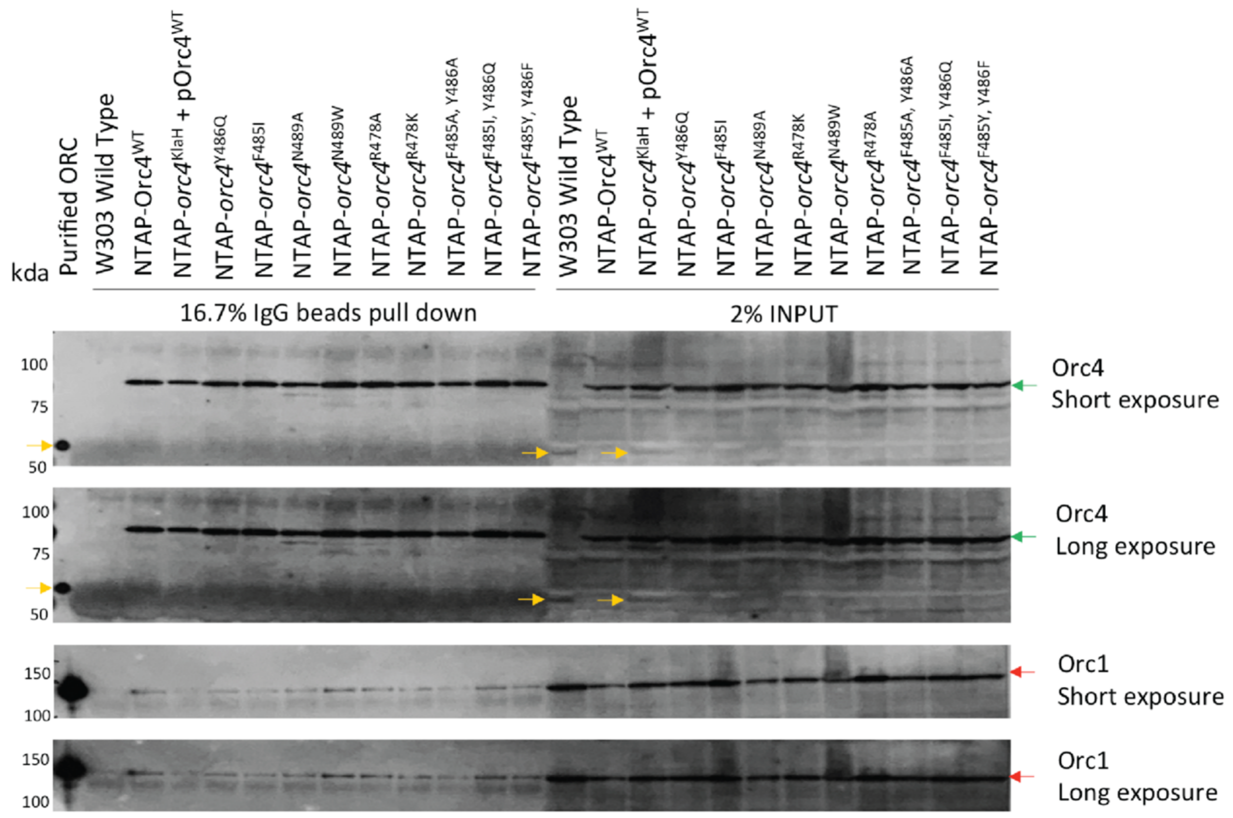

Supplementary Fig. 4 | Orc4 protein expression detection and ORC complex formation detection. Details of method is described in Method, Cell extract preparation, immunoprecipitation, immunoblot analysis and antibodies. NTAP-tagged Orc4 were immunoprecipitated via incubation with IgG beads. Wild type W303 strain, which contains non-tagged Orc4, is used parallelly as a control of pulldown assay. 2% of input and 16.7% of pulled-down lysate were loaded and subsequently immunoblot with anti-Orc4 (SB12) and anti-Orc1 (SB13). Purified ORC complex (including Orc1 and non-tagged Orc4) was also loaded as control for immunoblotting of Orc1 and Orc4. NTAP-tagged Orc4 is around 83kDa (indicated with green arrows) and non-tagged Orc4 is around 56kDa (indicated with yellow arrows), while Orc1 is around 120kDa (indicated with red arrows). Both short and long exposure of blots are indicated. Source data are provided as a Source Data file.

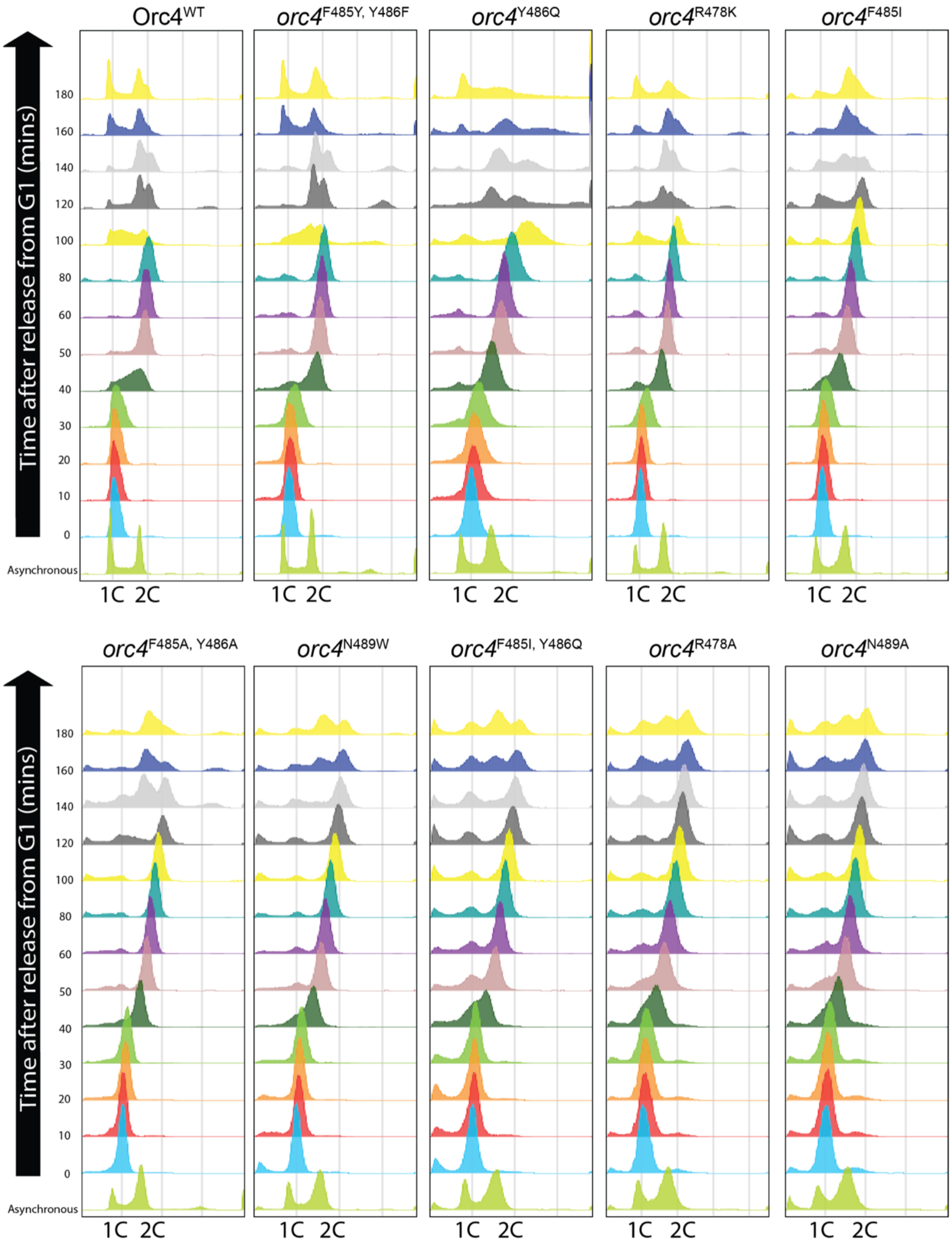

Supplementary Fig. 5 | Cell cycle of NTAP-Orc4 integrated strains. Flow cytometry was done by growing cells into log phase, arresting at G1 phase with α-factor block for 3 hours (around 1~2

cell cycle time length) and then releasing into S phase for different time point (as indicated above on the left). Different time points were harvest and prepared for flow cytometry with method previously described[50]. DNA strained with SYBR green. *Orc4* mutants seemed to have hard time going through S phase and progression through mitosis.

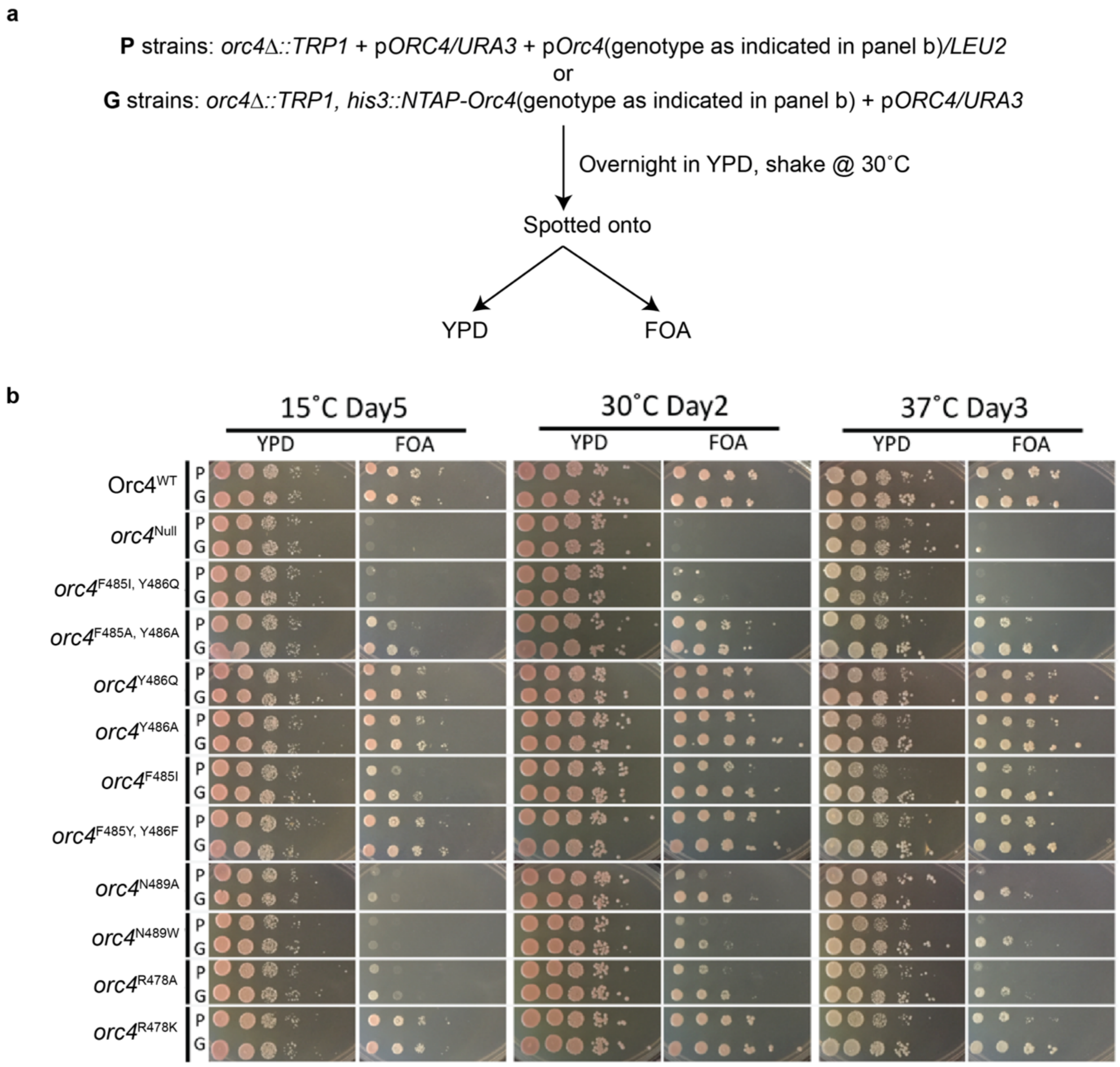

Supplementary Fig. 6 | Analysis of plasmid borne and genome integrated *Orc4* mutants. a, Schematic diagram of viability comparison assay between strains surviving dependent on single episomal origin (*Orc4* on plasmid, denoted as [P]) or multiple chromosomal origins (*Orc4* integrated into genome, denoted as [G]). The [P] strain relies on a CEN-based plasmid with a single replication origin to carry the tested Orc4 mutation and is therefore stringent. **b,** [P] strains (*orc4::TRP1* + p*ORC4/URA3* + p*orc4/LEU2*) and [G] strains (*his3::NTAP-orc4*^{mut}, *orc4::TRP1*, *bar1*Δ*::TRP1*, *LEU2::BrdU-Inc* + p*Orc4/URA3*) were grown overnight in YPD and spotted onto 5-FOA plates with 10-fold serial dilutions starting from 1.5×10^7 cells and spotted onto YPD plates as control. As controls for [P] strains, strain (*orc4::TRP1* + p*ORC4/URA3* + p*ORC4/LEU2*) and strain (*orc4::TRP1* + p*ORC4/URA3* + p*orc4*^{null}*/LEU2*) were spotted. As controls for [G] strains, strain (*his3::NTAP-Orc4*^{WT}, *orc4::TRP1*, *bar1*Δ*::TRP1*, *LEU2::BrdU-Inc* + p*Orc4/URA3*) and strain (*orc4::TRP1*, *bar1*Δ*::TRP1*, *LEU2::BrdU-Inc* + p*Orc4/URA3*) were spotted. Plates were cultured under 25˚C, 30˚C, or 37˚C for different days (as indicated) to test their temperature sensitivity. The strain lacking a NTAP-tagged Orc4 did not grow on FOA. The viability deficient

phenotype of *Orc4* mutants on single-origin plasmid seemed to be partially rescued when the mutants are integrated into the genome and survive on multiple origins.

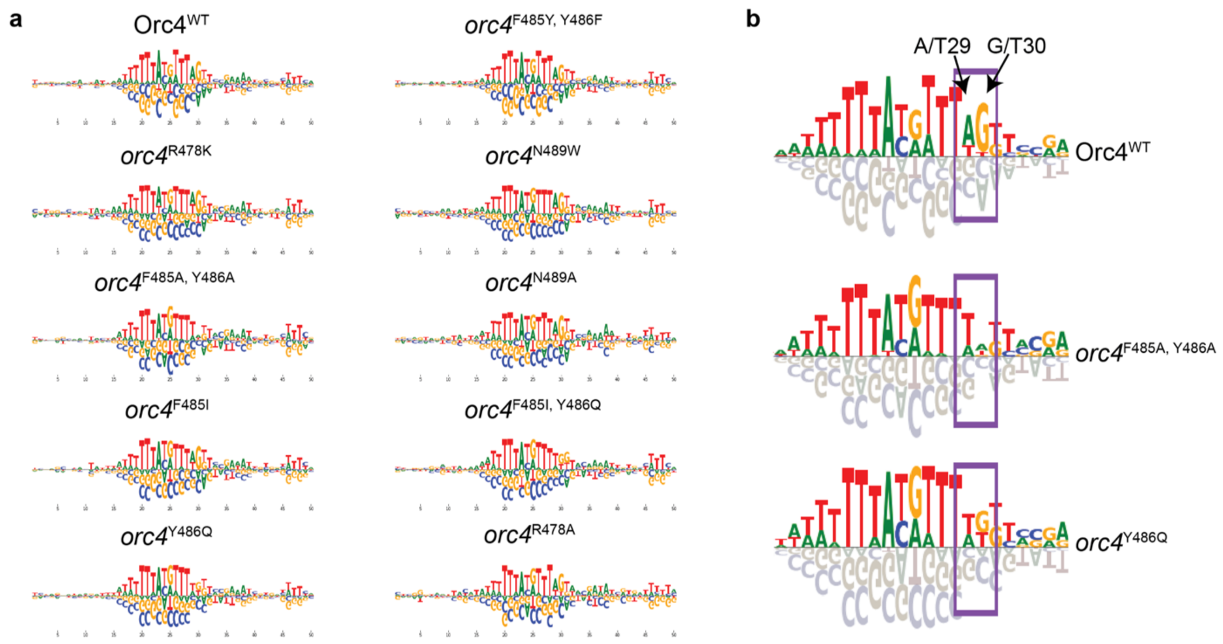

Supplementary Fig. 7 | ARS motif logos generated from MPOS assay using *ARS317* mutations. a, ARS motif logos for *Orc4* integrated strains at A and B1 elements generated using mutation library with *ARS317* sequence backbone. Same as Fig 3, top-half of logos representing the origin sequences that were selected-for in MPOS assay and bottom-half of logos representing the origin sequences that were selected-against in MPOS assay. **b,** Magnified view of A element region in a logo from *Orc4*^{WT}, *orc4*$^{F485A,\ Y486A}$, *orc4*^{Y486Q} strains with bottom-half of logo faded. Dark purple circles indicate the major changes at A/T29, G/T30 logo positions in the *Orc4* mutant strains.

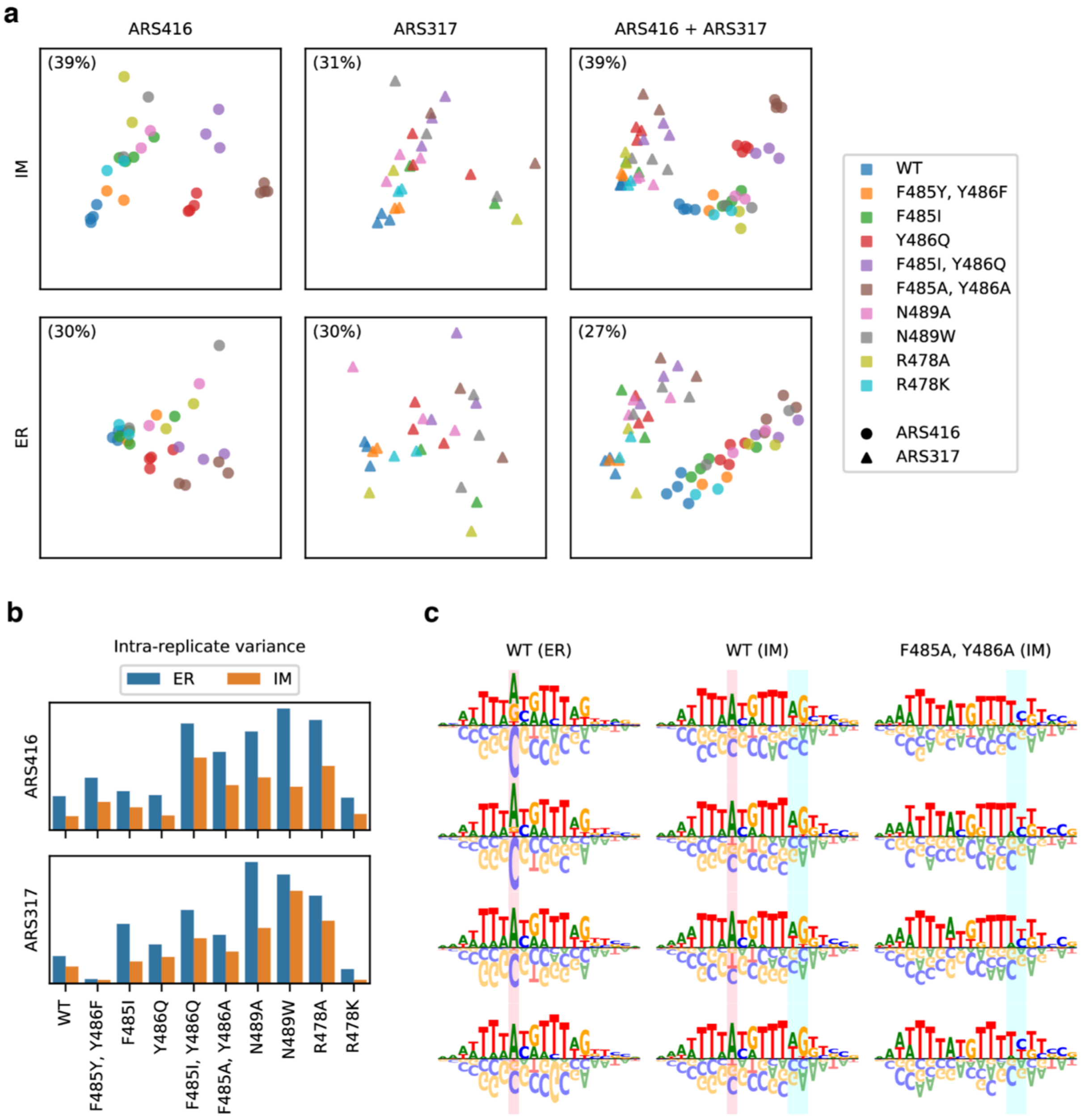

Supplementary Fig. 8 | Principal component analysis and comparison of motif inference methods. a, PCA analyses of motifs (performed on the *ARS416* library MPOS data, the *ARS317* library MPOS data, or both libraries), and inferred using either information maximization (IM) or enrichment ratios (ER). The variance explained by the first two principal components, corresponding to the x- and y-axes of each plot, is indicated in the upper left corner. The dots within each plot represent biologically independent MPOS experiments, dot color indicates the *Orc4* variant assayed, and dot shape indicates the library of mutated ARSs used as input. Note that motifs cluster according to the *Orc4* variant assayed, and that this clustering is stronger for information maximization (IM)-inferred motifs compared to enrichment ratio (ER)-inferred motifs. **b,** Total variance across the motifs inferred for each *Orc4* variant using either ER or IM inference. IM inference consistently yielded less intra-replicate variance than ER inference (48.3%

less on average for *ARS416* motifs and 39.9% less for *ARS317* motifs). This again reflects the robustness of IM inference in the face of experiment-to-experiment variation. **c,** Logos showing the *ARS416* motifs for two *Orc4* variants. For clarity, only 20 bp encompassing the essential A element are shown. ER motifs exhibited substantially more variability at key positions than did IM motifs (e.g. rose highlighted positions). *Orc4* mutants resulted in consistently and clearly visible differences in the inferred IM motifs (e.g. cyan positions).

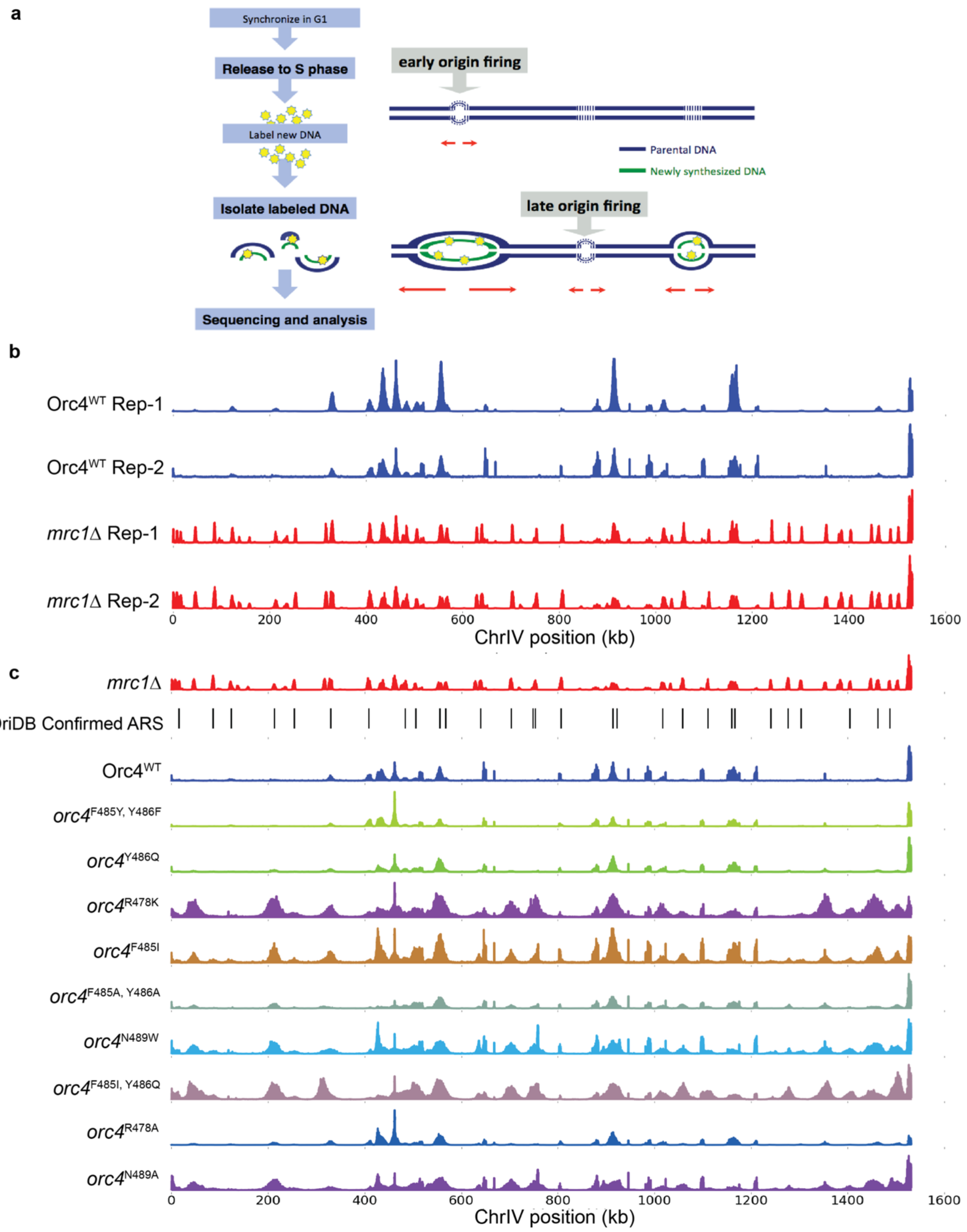

Supplementary Fig. 9 | Replicates of genome-wide replication origin profile. a, Schematic diagram for genome-wide replication origin profile analysis. Details of method is described in Methods, Genome-wide replication origin profile analysis. Briefly, Yeast cells were α-factor

blocked in G1 phase for 3 hours and then released into the growth medium (YPD with 200mM HU, 500uM EdU and 0.2mg/ml pronase E) for 90mins before harvest. Flow cytometry was done to check the stage of the cells. DNA is isolated from the harvested cells and sonicated using Bioruptor. EdU labeled newly synthesized DNA is pulldown by Click-iT chemistry with biotinylated azide and Streptavidin T1 magnetic beads. Then Illumina TruSeq Kit is used to establish and amplify the sequencing library. Sequencing data is then analyzed to show peaks on newly synthesized DNA with detailed computational method in Method, Computational analyses of replication origin profile and ChIP-seq data. **b-c,** Replicates of origin firing profiles in Fig. 3. Chromosome IV(ChrIV) is used as representation and replicates are from two independent experiments. **b** shows the direct comparison of two replicates with profiles from *Orc4*^{WT} and *mrc1*Δ strains shown as examples. **c** shows the genome-wide replication origin firing profiles from the all strains in replicate experiment.

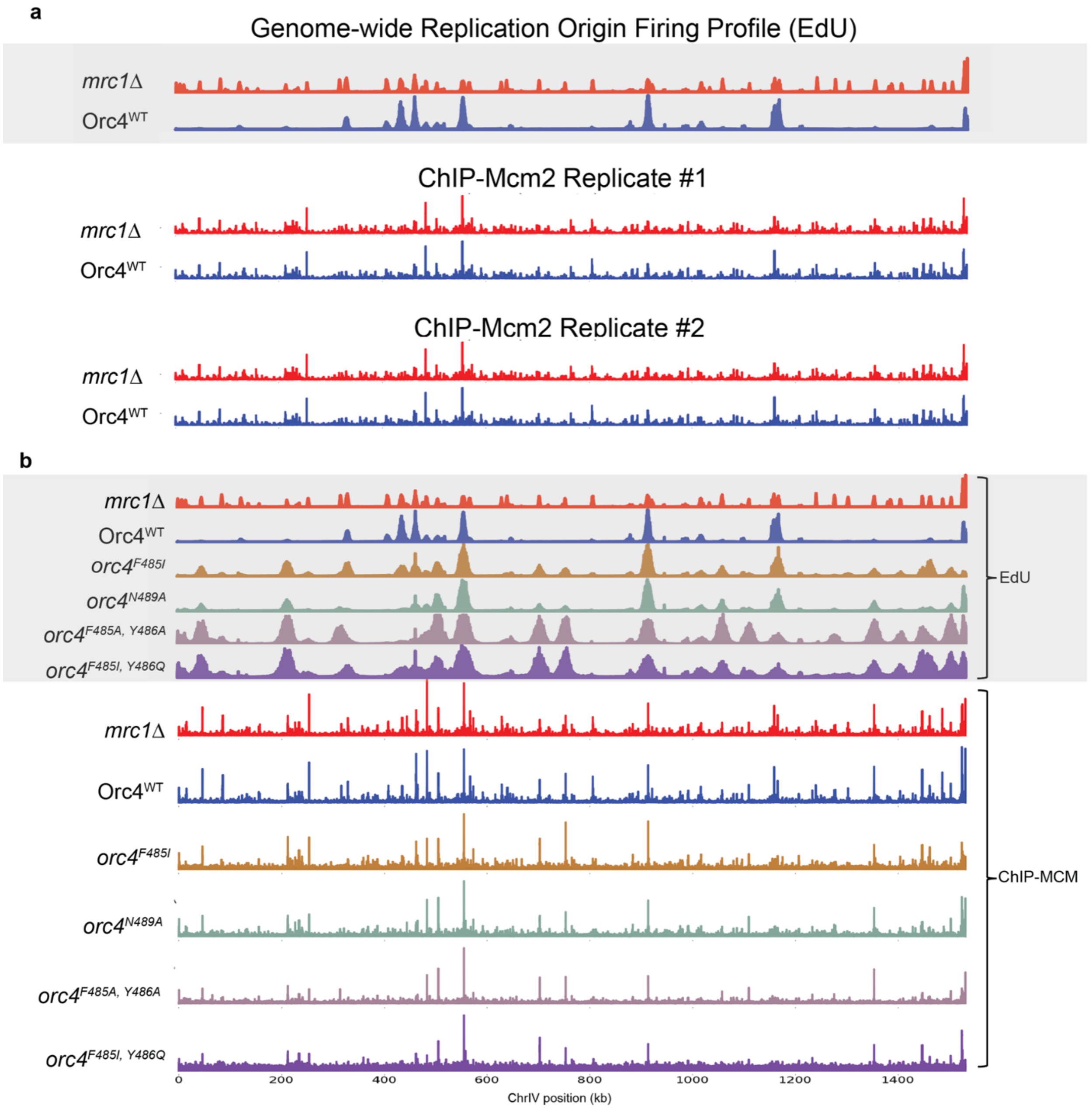

Supplementary Fig. 10 | Chromatin immuno-precipitation (ChIP) of MCM in *Orc4* strains. a-b, ChIP profile of MCM (anti-Mcm2) in G1 phase (*Orc4*^{WT}, *orc4*$^{F485I, Y486Q}$ and *mrc1*Δ profiles in Fig. 3). Chromosome IV(ChrIV) is used as representation. Genome-wide replication origin firing profiles from Fig. 3 is attached for better reference of origin firing pattern and are shadowed in grey. **a** shows the direct comparison of two independent replicates with Mcm2-ChIP profiles from *Orc4*^{WT} and *mrc1*Δ strains shown as examples. **b** shows the Mcm2-ChIP profiles of the all the strains from one of the two replicates.

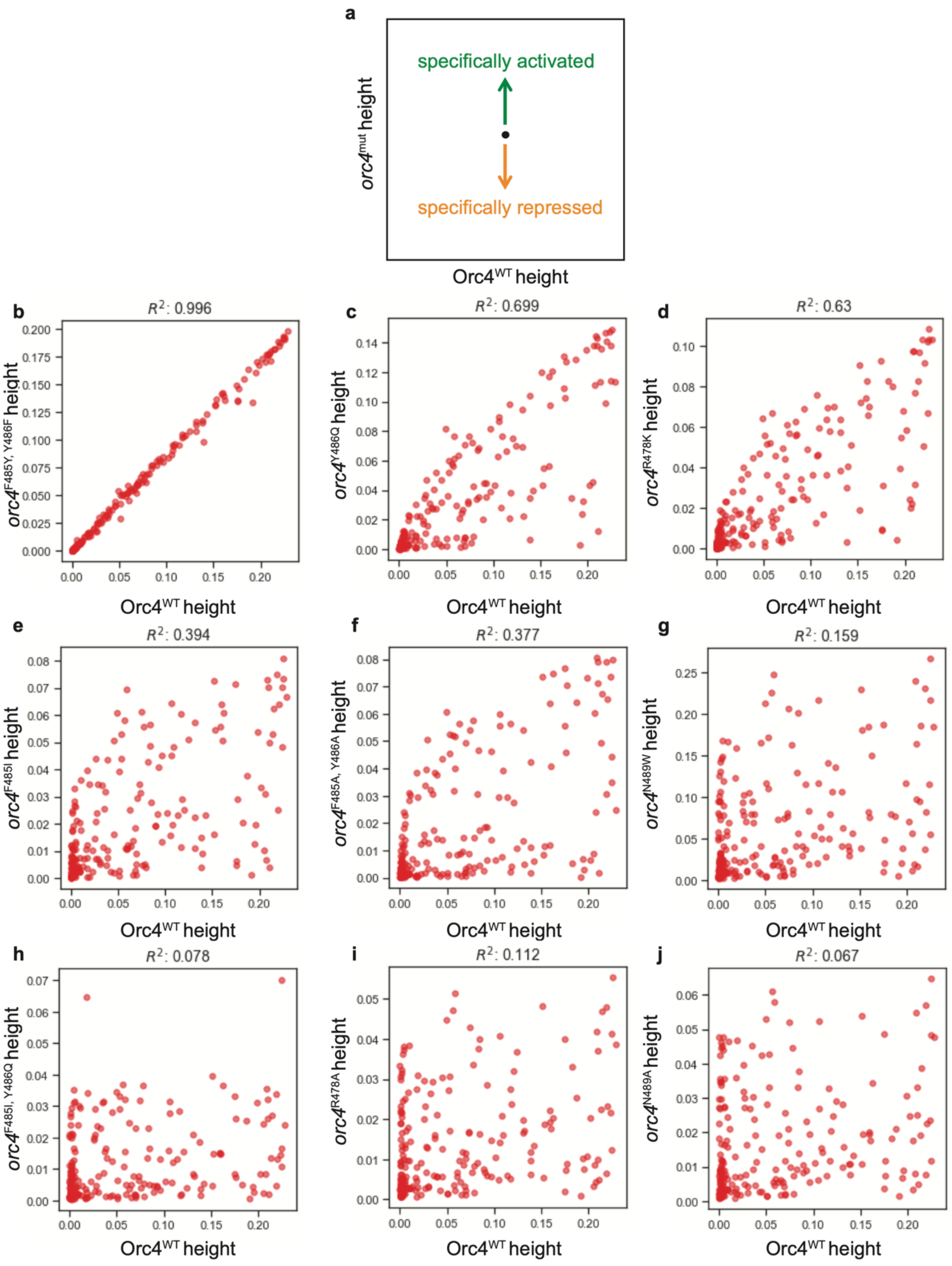
a
specifically activated
specifically repressed
orc4mut height
Orc4WT height
b
R2: 0.996
orc4F485Y, Y486F height
c
R2: 0.699
orc4Y486Q height
d
R2: 0.63
orc4R478K height
e
R2: 0.394
orc4F485I height
f
R2: 0.377
orc4F485A, Y486A height
g
R2: 0.159
orc4N489W height
h
R2: 0.078
orc4F485I, Y486Q height
i
R2: 0.112
orc4R478A height
j
R2: 0.067
orc4N489A height
Orc4WT height

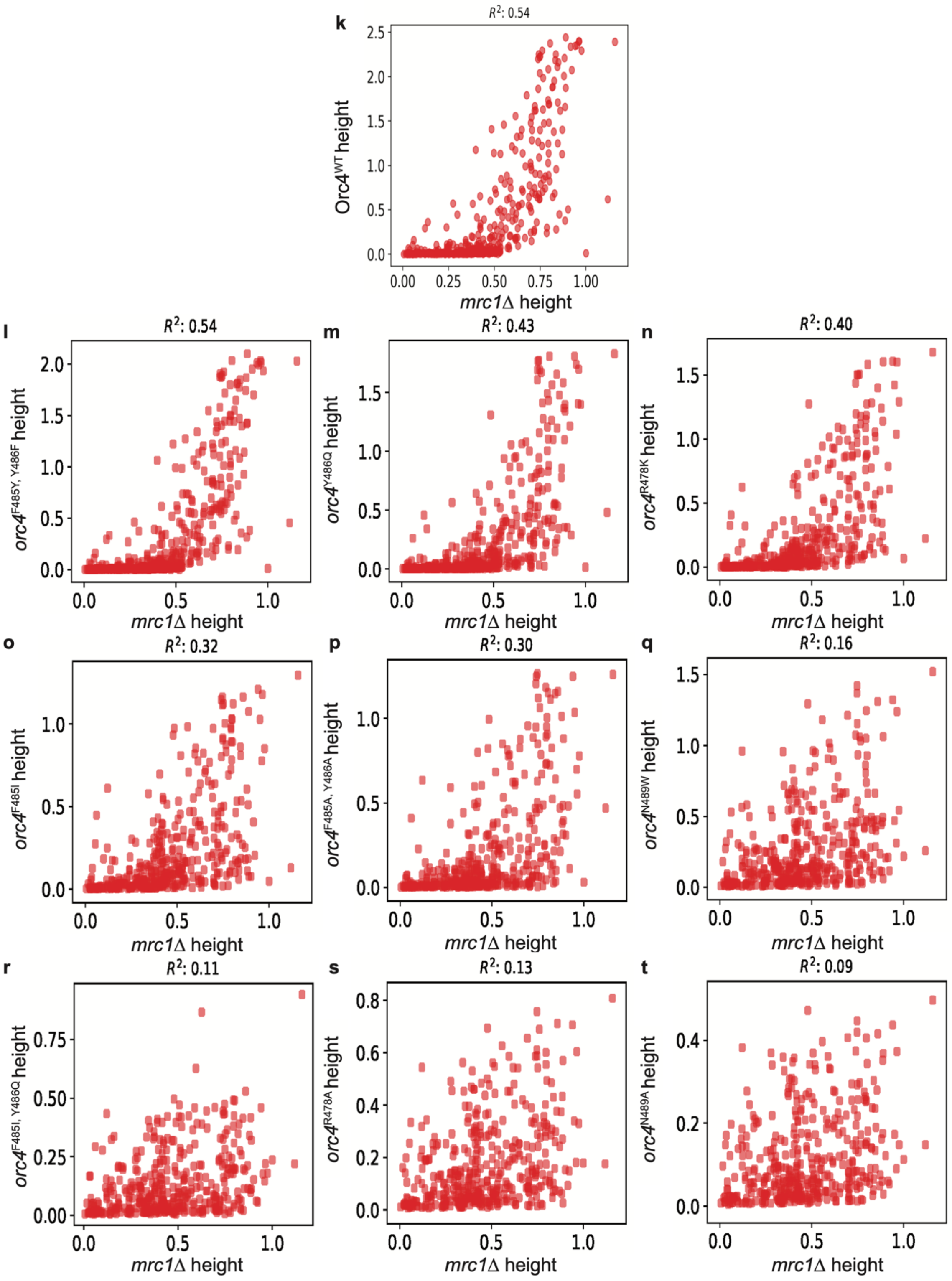

Supplementary Fig. 11 | Genomic origin firing peak heights scatter plot comparisons. Each dot represent a single replication origin that has its origin firing peak height in *Orc4^{WT}* (in **b-j**) or *mrc1*Δ (in **k-t**) as the x-value and its origin firing peak height in *orc4^{mut}* (in **b-j**, **l-t**) or *Orc4^{WT}* (in **k**) as y-value. Two origins exhibited aberrantly large height values, believed to have arisen from read mapping artefacts, and were removed from this analysis. Height values are normalized by

computing the number of reads bounding 99.5% of positions within each profile and divided the entire profile by this number. Coefficient of determination values (R^2) are shown atop each panel. **a**, Illustration diagram for **b**-**j** showing the directions of activation (in green) and repression (in orange) for each replication origin (denote as black dot) in *orc4* mutant strains. **b**-**j**, $orc4^{\mathrm{mut}}$ strains direct comparison with $Orc4^{WT}$ strain. **k**-**t**, all ten *Orc4* strains direct comparison with *mrc1*Δ strain.

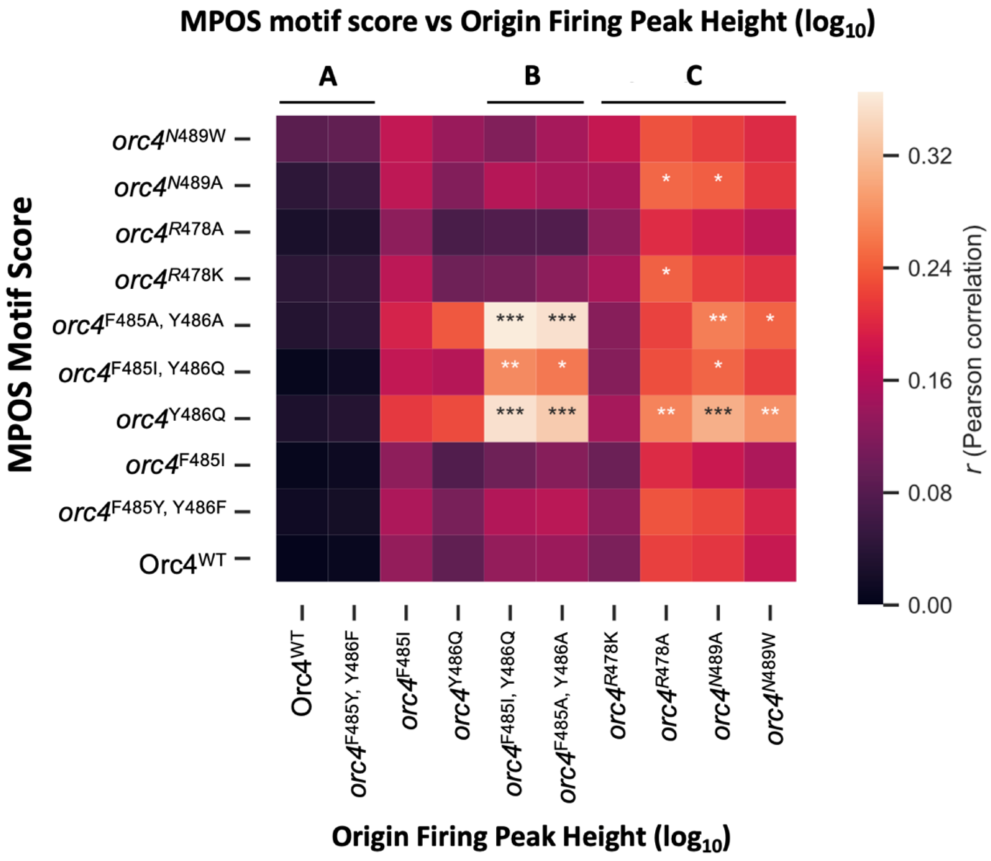

Supplementary Fig. 12 | Correlation of genomic origin peak height and MPOS motif scores. DNA sequences under origin firing peaks that were predicted to be ACS were obtained from OriDB[13,37] and used for analysis. MPOS motif scores were assigned by how good the annotated ACSs matched to the MPOS motifs. Correlations between origin firing peak heights (in $\log_{10}$) and MPOS motif scores were evaluated for each annotated ACS. A section indicates the origin peak heights in wild-type strain or wild-type like *orc4* mutant control strain. We took late vs early origin factors into consideration. However, a large value in origin height does not guarantee a high MPOS motif score. B section indicates the *orc4* F485 and Y486 mutants that were shown to have their origin sequence recognition altered (Fig. 2c and Fig. 4). C section indicates the *orc4* R478 and N489 mutation strains. P-values were also computed to assess the null hypothesis that log EdU heights and motif scores are not correlated; all P-values were Bonferoni corrected (by multiplying by the total number of tests). Significant correlations were indicated: *p<0.05, **p<0.01, ***p<0.001.

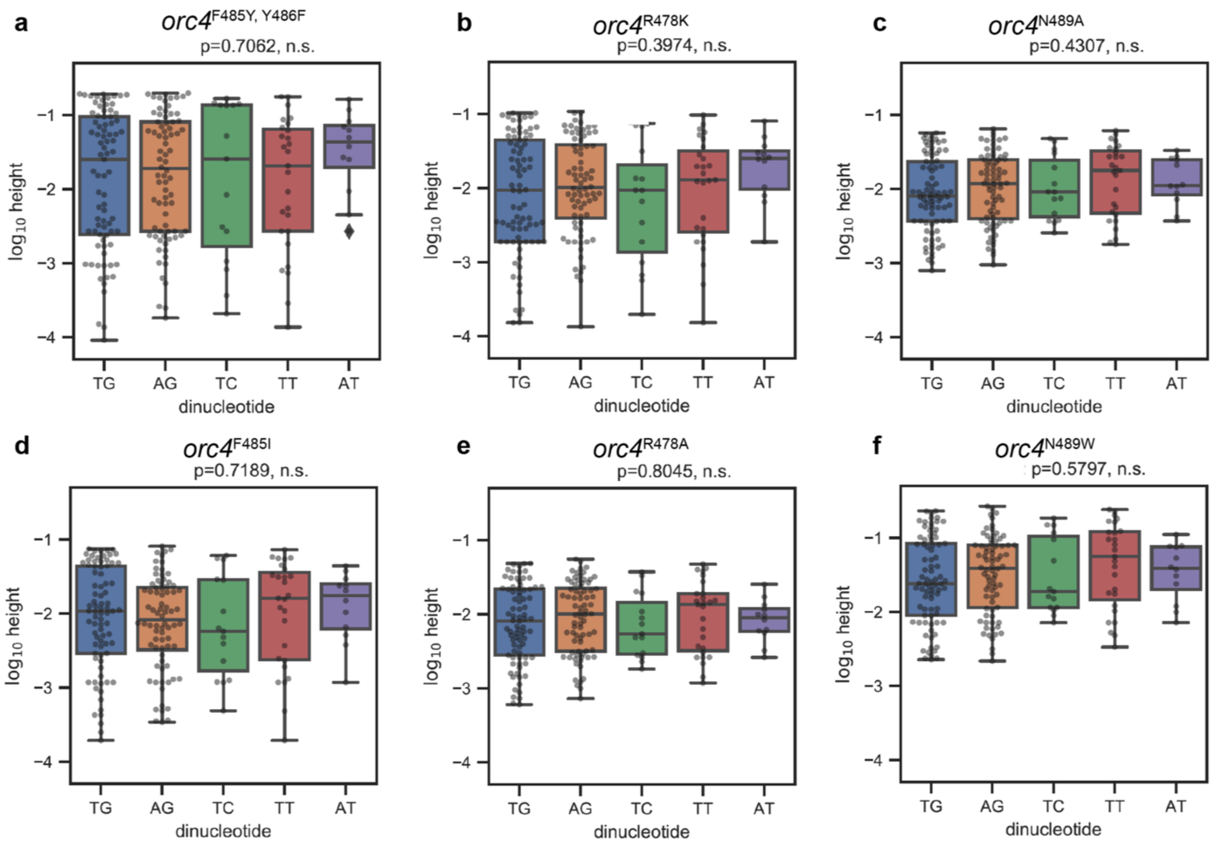

Supplementary Fig. 13 | Non-Y486 *orc4* mutants efficiently use origins with the "AG" dinucleotide. Supplemental figure for Fig. 4. Box plots for the 6 *orc4* mutant strains that do not have Y486 changed, except for the *orc4*$^{F485Y, Y486F}$ strain that contains a conserved mutation whose strain grows similar to *Orc4*^{WT} and therefore is an exception of Y486 mutation strain. **a.** *orc4*^{F485Y,Y486F}, **b.** *orc4*^{R478K}, **c.** *orc4*^{N489A}, **d.** *orc4*^{F485I}, **e.** *orc4*^{R478A}, **f.** *orc4*^{N489W}. Y-axis is genomic origin firing peak heights in $\log_{10}$. Each dot denotes an annotated ACS. Box plots elements: the minimum height, first (lower) quartile, median, third (upper) quartile, and maximum height. Diamond denotes outliers that exhibited aberrantly large values.

Supplementary Tables

Orc4 Mutation	Phenotype
Klα-helix	# @30˚C (N=4)
Δα-helix	# @all three temperature (N=2)
F485I, Y486Q	*** @30˚C; # @15˚C (CS); # @37˚C (TS) (N=3)
N489W	*** @30˚C; # @15˚C (CS); # @37˚C (TS) (N=3)
N489A	*** @30˚C; # @15˚C (CS); # @37˚C (TS) (N=3)
R478A	** @30˚C; *** @15˚C (CS); # @37˚C (TS) (N=3)
F485I	* @30˚C; ** @15˚C (CS); * @37˚C (N=3)
F485A, Y486A	* @30˚C; ** @15˚C (CS); * @37˚C (N=3)
Y490L	* @30˚C; ** @15˚C (CS); * @37˚C (N=2)
F492S	* @30˚C; ** @15˚C (CS); * @37˚C (N=2)
R478K	- @all three temperature (N=3)
Y486Q	- @all three temperature (N=3)
F485A	~ @30˚C; - @15˚C; ~ @37˚C (N=3)
Y486A	~ @30˚C; - @15˚C; ~ @37˚C (N=3)
Y490A	~ @30˚C; - @15˚C; ~ @37˚C (N=3)
F492A	~ @30˚C; - @15˚C; ~ @37˚C (N=3)
Q493R	~ @all three temperature (N=2)
D479R	~ @all three temperature (N=2)
V226A	~ @all three temperature (N=2)
V226R	~ @all three temperature (N=2)
R227A	~ @all three temperature (N=2)
Q493A	~ @all three temperature (N=3)
K402A	~ @all three temperature (N=3)
K402E	~ @all three temperature (N=3)
T482D	~ @all three temperature (N=5)
T482R	~ @all three temperature (N=3)
T482A	~ @all three temperature (N=3)
T482I	~ @all three temperature (N=3)
F485Y, Y486F	~ @all three temperature (N=3)
Y490R	~ @all three temperature (N=3)
F492L	~ @all three temperature (N=3)
R227D	~ @all three temperature (N=3)

Symbols denotes viability deficient phenotypes. ~ denotes minimal or not deficient; - denotes slightly deficient; * denotes moderate deficient; ** denotes strong deficient; *** denotes severe deficient; # denotes lethal

Supplementary Table 1 | Summary of plasmid shuffle assay *Orc4* mutant phenotypes. *Orc4* mutant viability phenotypes summarized from plasmid shuffle assay (Supplementary Fig. 1). Indicated symbols denotes different viability phenotypes.

Orc2 Mutation	Phenotype
Δ390-398	# @all three temperature (N=2)
Δ393-398	# @all three temperature (N=2)
W396A	# @all three temperature (N=3)
Y395A	# @all three temperature (N=2)
N398A	** @30˚C; # @15˚C (CS); # @37˚C (TS) (N=2)
T393A	~ @all three temperature (N=3)
K394A	~ @all three temperature (N=2)

Symbols denotes viability deficient phenotypes. ~ denotes minimal or not deficient; - denotes slightly deficient; * denotes moderate deficient; ** denotes strong deficient; *** denotes severe deficient; # denotes lethal

Supplementary Table 2 | Summary of plasmid shuffle assay *Orc2* mutant phenotypes. *Orc2* mutant viability phenotypes summarized from plasmid shuffle assay (Supplementary Fig. 2). Indicated symbols denotes different viability phenotypes.

Orc4 Integrated Strains	Doubling Time
WT	87mins
F485Y, Y486F	89mins
Y486Q	91mins
R478K	91.5mins
F485I	93mins
F485A, Y486A	97mins
N489W	112mins
F485I, Y486Q	127mins
R478A	132mins
N489A	134.5mins

Supplementary Table 3 | Summary of doubling time of NTAP-Orc4 integrated strains.
Doubling time were calculated based on growth curves (see Fig. 1f) log phase cell concentration.

$$k = \frac{\Delta \log(Cell\ Conc.)}{\Delta Time} = \frac{\log(2)}{Doubling\ Time} \quad (4)$$

$$Doubling\ Time = \log(2) / k \quad (5)$$

k dictates the slope of linear regression line of growth curves log phase.

Supplementary Methods

Strain	Genotype	Source
**YB51**	*MATα orc4::TRP1 ade2-1 ura3-1 his 3-11, 15 trp1-1 leu2-3,112 can1-100 + pRS415/ORC4*	This lab
**YS856**	*MATa ade2-1 ura3-1 his3-11, 15 trp1-1 leu2-3,112 can1-100*	This study
**YB410**	*MATα orc2::TRP1 ade2-1 ura3-1 his 3-11, 15 trp1-1 leu2-3,112 can1-100 + pRS316/ORC2*	This lab
**YS3285**	*MATa mrc1Δ::KanMX6 bar1Δ::TRP1 URA3::BrdU-Inc ade2-1 ura3-1 his3-11, 15 trp1-1 leu2-3,112 can1-100*	This study
**YB 1519**	*MATα orc4::TRP1 ade2-1 ura3-1 his 3-11, 15 trp1-1 leu2-3,112 can1-100 + pRS416/ORC4 + pRS415/ORC4*	This study
**YB 1515**	*MATα orc4::TRP1 ade2-1 ura3-1 his 3-11, 15 trp1-1 leu2-3,112 can1-100 + pRS416/ORC4 + pRS415*	This study
**YB1639**	*MATα orc4::TRP1 ade2-1 ura3-1 his 3-11, 15 trp1-1 leu2-3,112 can1-100 + pRS416/ORC4 + pRS415/orc4Δα-helix*	This study
**YB 1514**	*MATα orc4::TRP1 ade2-1 ura3-1 his 3-11, 15 trp1-1 leu2-3,112 can1-100 + pRS416/ORC4 + pRS415/orc4Klα-helix*	This study
**YB 1520**	*MATα orc4::TRP1 ade2-1 ura3-1 his 3-11, 15 trp1-1 leu2-3,112 can1-100 + pRS416/ORC4 + pRS415/orc4Y486Q*	This study
**YB 1521**	*MATα orc4::TRP1 ade2-1 ura3-1 his 3-11, 15 trp1-1 leu2-3,112 can1-100 + pRS416/ORC4 + pRS415/orc4Y486A*	This study
**YB 1522**	*MATα orc4::TRP1 ade2-1 ura3-1 his 3-11, 15 trp1-1 leu2-3,112 can1-100 + pRS416/ORC4 + pRS415/orc4F485I*	This study
**YB 1523**	*MATα orc4::TRP1 ade2-1 ura3-1 his 3-11, 15 trp1-1 leu2-3,112 can1-100 + pRS416/ORC4 + pRS415/orc4F485A*	This study
**YB 1524**	*MATα orc4::TRP1 ade2-1 ura3-1 his 3-11, 15 trp1-1 leu2-3,112 can1-100 + pRS416/ORC4 + pRS415/orc4N489A*	This study
**YB 1525**	*MATα orc4::TRP1 ade2-1 ura3-1 his 3-11, 15 trp1-1 leu2-3,112 can1-100 + pRS416/ORC4 + pRS415/orc4N489W*	This study
**YB 1526**	*MATα orc4::TRP1 ade2-1 ura3-1 his 3-11, 15 trp1-1 leu2-3,112 can1-100 + pRS416/ORC4 + pRS415/orc4F492A*	This study
**YB 1527**	*MATα orc4::TRP1 ade2-1 ura3-1 his 3-11, 15 trp1-1 leu2-3,112 can1-100 + pRS416/ORC4 + pRS415/orc4Y490A*	This study
**YB 1528**	*MATα orc4::TRP1 ade2-1 ura3-1 his 3-11, 15 trp1-1 leu2-3,112 can1-100 + pRS416/ORC4 + pRS415/orc4R478A*	This study
**YB 1555**	*MATα orc4::TRP1 ade2-1 ura3-1 his 3-11, 15 trp1-1 leu2-3,112 can1-100 + pRS416/ORC4 + pRS415/orc4Q493A*	This study
**YB 1556**	*MATα orc4::TRP1 ade2-1 ura3-1 his 3-11, 15 trp1-1 leu2-3,112 can1-100 + pRS416/ORC4 + pRS415/orc4K402A*	This study
**YB 1557**	*MATα orc4::TRP1 ade2-1 ura3-1 his 3-11, 15 trp1-1 leu2-3,112 can1-100 + pRS416/ORC4 + pRS415/orc4K402E*	This study
**YB 1558**	*MATα orc4::TRP1 ade2-1 ura3-1 his 3-11, 15 trp1-1 leu2-3,112 can1-100 + pRS416/ORC4 + pRS415/orc4T482A*	This study
**YB 1559**	*MATα orc4::TRP1 ade2-1 ura3-1 his 3-11, 15 trp1-1 leu2-3,112 can1-100 + pRS416/ORC4 + pRS415/orc4T482R*	This study

**YB 1560**	*MATα orc4::TRP1 ade2-1 ura3-1 his 3-11, 15 trp1-1 leu2-3,112 can1-100 + pRS416/ORC4 + pRS415/orc4T482I*	This study
**YB 1571**	*MATα orc4::TRP1 ade2-1 ura3-1 his 3-11, 15 trp1-1 leu2-3,112 can1-100 + pRS416/ORC4 + pRS415/orc4T482D*	This study
**YB 1562**	*MATα orc4::TRP1 ade2-1 ura3-1 his 3-11, 15 trp1-1 leu2-3,112 can1-100 + pRS416/ORC4 + pRS415/orc4R478K*	This study
**YB 1563**	*MATα orc4::TRP1 ade2-1 ura3-1 his 3-11, 15 trp1-1 leu2-3,112 can1-100 + pRS416/ORC4 + pRS415/orc4F485A, Y486A*	This study
**YB 1564**	*MATα orc4::TRP1 ade2-1 ura3-1 his 3-11, 15 trp1-1 leu2-3,112 can1-100 + pRS416/ORC4 + pRS415/orc4F485Y, Y486F*	This study
**YB1635**	*MATα orc4::TRP1 ade2-1 ura3-1 his 3-11, 15 trp1-1 leu2-3,112 can1-100 + pRS416/ORC4 + pRS415/orc4F485I, Y486Q*	This study
**YKC01**	*MATα orc4::TRP1 ade2-1 ura3-1 his 3-11, 15 trp1-1 leu2-3,112 can1-100 + pRS416/ORC4 + pRS415/orc4F492L*	This study
**YKC02**	*MATα orc4::TRP1 ade2-1 ura3-1 his 3-11, 15 trp1-1 leu2-3,112 can1-100 + pRS416/ORC4 + pRS415/orc4Y490L*	This study
**YKC03**	*MATα orc4::TRP1 ade2-1 ura3-1 his 3-11, 15 trp1-1 leu2-3,112 can1-100 + pRS416/ORC4 + pRS415/orc4Y490R*	This study
**YKC04**	*MATα orc4::TRP1 ade2-1 ura3-1 his 3-11, 15 trp1-1 leu2-3,112 can1-100 + pRS416/ORC4 + pRS415/orc4F492S*	This study
**YB1637**	*MATα orc4::TRP1 ade2-1 ura3-1 his 3-11, 15 trp1-1 leu2-3,112 can1-100 + pRS416/ORC4 + pRS415/orc4R227D*	This study
**YEH003**	*MATα orc4::TRP1 ade2-1 ura3-1 his 3-11, 15 trp1-1 leu2-3,112 can1-100 + pRS416/ORC4 + pRS415/orc4V226A*	This study
**YEH004**	*MATα orc4::TRP1 ade2-1 ura3-1 his 3-11, 15 trp1-1 leu2-3,112 can1-100 + pRS416/ORC4 + pRS415/orc4V226R*	This study
**YEH005**	*MATα orc4::TRP1 ade2-1 ura3-1 his 3-11, 15 trp1-1 leu2-3,112 can1-100 + pRS416/ORC4 + pRS415/orc4R227A*	This study
**YEH002**	*MATα orc4::TRP1 ade2-1 ura3-1 his 3-11, 15 trp1-1 leu2-3,112 can1-100 + pRS416/ORC4 + pRS415/orc4Q493R*	This study
**YEH001**	*MATα orc4::TRP1 ade2-1 ura3-1 his 3-11, 15 trp1-1 leu2-3,112 can1-100 + pRS416/ORC4 + pRS415/orc4D479R*	This study
**YS2251**	*MATa bar1Δ::TRP1 ade2-1 ura3-1 his3-11, 15 trp1-1 leu2-3,112 can1-100*	
**YB 1549**	*MATa bar1Δ::TRP1 LEU2::BrdU-Inc ade2-1 ura3-1 his3-11, 15 trp1-1 leu2-3,112 can1-100*	This study
**YB1588**	*MATa orc4::TRP1 bar1Δ::TRP1 LEU2::BrdU-Inc ade2-1 ura3-1 his3-11, 15 trp1-1 leu2-3,112 can1-100 + pRS416/ORC4*	This study
**YB 1623**	*MATa his3::TAP-ORC4 orc4::TRP1 bar1Δ::TRP1 LEU2::BrdU-Inc ade2-1 ura3-1 his3-11, 15 trp1-1 leu2-3,112 can1-100 + pRS416/ORC4*	This study
**YB 1624**	*MATa his3::TAP-orc4F485I orc4::TRP1 bar1Δ::TRP1 LEU2::BrdU-Inc ade2-1 ura3-1 his3-11, 15 trp1-1 leu2-3,112 can1-100 + pRS416/ORC4*	This study

**YB 1625**	*MATa his3::TAP-orc4N489A orc4::TRP1 bar1Δ::TRP1 LEU2::BrdU-Inc ade2-1 ura3-1 his3-11, 15 trp1-1 leu2-3,112 can1-100 + pRS416/ORC4*	This study
**YB 1630**	*MATa his3::TAP-orc4KLaH orc4::TRP1 bar1Δ::TRP1 LEU2::BrdU-Inc ade2-1 ura3-1 his3-11, 15 trp1-1 leu2-3,112 can1-100 + pRS416/ORC4*	This study
**YB 1631**	*MATa his3::TAP-orc4F485Y+Y486F orc4::TRP1 bar1Δ::TRP1 LEU2::BrdU-Inc ade2-1 ura3-1 his3-11, 15 trp1-1 leu2-3,112 can1-100 + pRS416/ORC4*	This study
**YB 1632**	*MATa his3::TAP-orc4R478K orc4::TRP1 bar1Δ::TRP1 LEU2::BrdU-Inc ade2-1 ura3-1 his3-11, 15 trp1-1 leu2-3,112 can1-100 + pRS416/ORC4*	This study
**YB 1633**	*MATa his3::TAP-orc4ΔaH orc4::TRP1 bar1Δ::TRP1 LEU2::BrdU-Inc ade2-1 ura3-1 his3-11, 15 trp1-1 leu2-3,112 can1-100 + pRS416/ORC4*	This study
**YB1647**	*MATa his3::TAP-orc4N489W orc4::TRP1 bar1Δ::TRP1 LEU2::BrdU-Inc ade2-1 ura3-1 his3-11, 15 trp1-1 leu2-3,112 can1-100 + pRS416/ORC4*	This study
**YB1648**	*MATa his3::TAP-orc4R478A orc4::TRP1 bar1Δ::TRP1 LEU2::BrdU-Inc ade2-1 ura3-1 his3-11, 15 trp1-1 leu2-3,112 can1-100 + pRS416/ORC4*	This study
**YB1652**	*MATa his3::TAP-orc4Y486Q orc4::TRP1 bar1Δ::TRP1 LEU2::BrdU-Inc ade2-1 ura3-1 his3-11, 15 trp1-1 leu2-3,112 can1-100 + pRS416/ORC4*	This study
**YB1653**	*MATa his3::TAP-orc4F485A+Y486A orc4::TRP1 bar1Δ::TRP1 LEU2::BrdU-Inc ade2-1 ura3-1 his3-11, 15 trp1-1 leu2-3,112 can1-100 + pRS416/ORC4*	This study
**YB1654**	*MATa his3::TAP-orc4F485I+Y486Q orc4::TRP1 bar1Δ::TRP1 LEU2::BrdU-Inc ade2-1 ura3-1 his3-11, 15 trp1-1 leu2-3,112 can1-100 + pRS416/ORC4*	This study
**YB1649**	*MATa his3::TAP-ORC4 orc4::TRP1 bar1Δ::TRP1 LEU2::BrdU-Inc ade2-1 ura3-1 his3-11, 15 trp1-1 leu2-3,112 can1-100*	This study
**YB1650**	*MATa his3::TAP-orc4F485I orc4::TRP1 bar1Δ::TRP1 LEU2::BrdU-Inc ade2-1 ura3-1 his3-11, 15 trp1-1 leu2-3,112 can1-100*	This study
**YB1655**	*MATa his3::TAP-orc4N489A orc4::TRP1 bar1Δ::TRP1 LEU2::BrdU-Inc ade2-1 ura3-1 his3-11, 15 trp1-1 leu2-3,112 can1-100*	This study
**YB1656**	*MATa his3::TAP-orc4F485Y+Y486F orc4::TRP1 bar1Δ::TRP1 LEU2::BrdU-Inc ade2-1 ura3-1 his3-11, 15 trp1-1 leu2-3,112 can1-100*	This study
**YB1657**	*MATa his3::TAP-orc4R478K orc4::TRP1 bar1Δ::TRP1 LEU2::BrdU-Inc ade2-1 ura3-1 his3-11, 15 trp1-1 leu2-3,112 can1-100*	This study

**YB1658**	*MATa his3::TAP-orc4N489W orc4::TRP1 bar1Δ::TRP1 LEU2::BrdU-Inc ade2-1 ura3-1 his3-11, 15 trp1-1 leu2-3,112 can1-100*	This study
**YB1659**	*MATa his3::TAP-orc4R478A orc4::TRP1 bar1Δ::TRP1 LEU2::BrdU-Inc ade2-1 ura3-1 his3-11, 15 trp1-1 leu2-3,112 can1-100*	This study
**YB1660**	*MATa his3::TAP-orc4Y486Q orc4::TRP1 bar1Δ::TRP1 LEU2::BrdU-Inc ade2-1 ura3-1 his3-11, 15 trp1-1 leu2-3,112 can1-100*	This study
**YB1661**	*MATa his3::TAP-orc4F485A+Y486A orc4::TRP1 bar1Δ::TRP1 LEU2::BrdU-Inc ade2-1 ura3-1 his3-11, 15 trp1-1 leu2-3,112 can1-100*	This study
**YB1662**	*MATa his3::TAP-orc4F485I+Y486Q orc4::TRP1 bar1Δ::TRP1 LEU2::BrdU-Inc ade2-1 ura3-1 his3-11, 15 trp1-1 leu2-3,112 can1-100*	This study
**YB 1620**	*MATα orc2::TRP1 ade2-1 ura3-1 his 3-11, 15 trp1-1 leu2-3,112 can1-100 + pRS316/ORC2 + pRS415*	This study
**YB 1619**	*MATα orc2::TRP1 ade2-1 ura3-1 his 3-11, 15 trp1-1 leu2-3,112 can1-100 + pRS316/ORC2 + pRS415/ORC2*	This study
**YB 1621**	*MATα orc2::TRP1 ade2-1 ura3-1 his 3-11, 15 trp1-1 leu2-3,112 can1-100 + pRS316/ORC2 + pRS415/orc2T393A*	This study
**YB 1611**	*MATα orc2::TRP1 ade2-1 ura3-1 his 3-11, 15 trp1-1 leu2-3,112 can1-100 + pRS316/ORC2 + pRS415/orc2W396A*	This study
**YKC05**	*MATα orc2::TRP1 ade2-1 ura3-1 his 3-11, 15 trp1-1 leu2-3,112 can1-100 + pRS316/ORC2 + pRS415/orc2K394A*	This study
**YKC06**	*MATα orc2::TRP1 ade2-1 ura3-1 his 3-11, 15 trp1-1 leu2-3,112 can1-100 + pRS316/ORC2 + pRS415/orc2Y395A*	This study
**YKC07**	*MATα orc2::TRP1 ade2-1 ura3-1 his 3-11, 15 trp1-1 leu2-3,112 can1-100 + pRS316/ORC2 + pRS415/orc2N398A*	This study
**YKC08**	*MATα orc2::TRP1 ade2-1 ura3-1 his 3-11, 15 trp1-1 leu2-3,112 can1-100 + pRS316/ORC2 + pRS415/orc2Δ393-398*	This study
**YKC09**	*MATα orc2::TRP1 ade2-1 ura3-1 his 3-11, 15 trp1-1 leu2-3,112 can1-100 + pRS316/ORC2 + pRS415/orc2Δ390-398*	This study

Supplementary Methods Table 1 | Genotype and resources of yeast strains used in this study

Primer Names	Primer Sequence	Note
**YEH001**	GCGACCGCAGCATTTcaaGCTAGCAATTATCAATTTCAGGG	Used for Orc4 mutagenesis
**YEH002**	CCCTGAAATTGATAATTGCTAGCttgAAATGCTGCGGTCGC	Used for Orc4 mutagenesis
**YEH003**	CGACCGCAGCATTTgccGCTAGCAATTATCAATTTCAG	Used for Orc4 mutagenesis
**YEH004**	CTGAAATTGATAATTGCTAGCggcAAATGCTGCGGTCGC	Used for Orc4 mutagenesis
**YEH005**	CGACCGCAGCAattTACGCTAGCAATTATCAATTTCAGGG	Used for Orc4 mutagenesis
**YEH006**	CCCTGAAATTGATAATTGCTAGCGTAaatTGCTGCGGTCG	Used for Orc4 mutagenesis
**YEH007**	CGACCGCAGCAgctTACGCTAGCAATTATCAATTTCAGGG	Used for Orc4 mutagenesis
**YEH008**	CCCTGAAATTGATAATTGCTAGCGTAagcTGCTGCGGTCG	Used for Orc4 mutagenesis
**YEH009**	CGACCGCAGCATTTTACGCTAGCgctTATCAATTTCAGGG	Used for Orc4 mutagenesis
**YEH010**	CCTGAAATTGATAagcGCTAGCGTAAAATGCTGCGGTCGC	Used for Orc4 mutagenesis
**YEH011**	GCATTTTACGCTAGCtggTATCAATTTCAGGGCACCATGATCCCG	Used for Orc4 mutagenesis

**YEH012**	CGGGATCATGGTGCCCTGAAATTGATAccaGCTAGCGTAAAATGC	Used for Orc4 mutagenesis
**YEH013**	ACCGCAGCATTTTACGCTAGCAATTATCAAgctCAGGGCACC	Used for Orc4 mutagenesis
**YEH014**	GGTGCCCTGagcTTGATAATTGCTAGCGTAAAATGCTGCGGT	Used for Orc4 mutagenesis
**YEH015**	ACCGCAGCATTTTACGCTAGCAATgctCAATTTCAGGGC	Used for Orc4 mutagenesis
**YEH016**	GCCCTGAAATTGagcATTGCTAGCGTAAAATGCTGCGGT	Used for Orc4 mutagenesis
**YEH017**	CCGAGAAATCAGCCGTTGGTTTGgcaGATAATGCGAC	Used for Orc4 mutagenesis
**YEH018**	GTCGCATTATCtgcCAAACCAACGGCTGATTTCTCGG	Used for Orc4 mutagenesis
**YEH024**	CCGTTGGTTTGAGAGATAATGAAGACTCTGCTATACAATCAGCAAACTACAATTCATCCGGCACCATGATCCCGTTTGAC	Used for Orc4 mutagenesis
**YEH025**	GTCAAACGGGATCATGGTGCCGGATGAATTGTAGTTTGCTGATTGTATAGCAGAGTCTTCATTATCTCTCAAACCAACGG	Used for Orc4 mutagenesis
**YEH026**	GCCGTTGGTTTGAGAGATAATGGCACCATGATCCCGTTTGAC	Used for Orc4 mutagenesis
**YEH027**	GTCAAACGGGATCATGGTGCCATTATCTCTCAAACCAACGGC	Used for Orc4 mutagenesis
**YEH032**	GCATTTTACGCTAGCAATTATCAATTTgccGGCACCATGATCCC	Used for Orc4

		mutagenesis
**YEH033**	GGGATCATGGTGCCggcAAATTGATAATTGCTAGCGTAAAATGC	Used for Orc4 mutagenesis
**YEH034**	GCATTTTACGCTAGCAATTATCAATTTagaGGCACCATGATCCCG	Used for Orc4 mutagenesis
**YEH035**	CGGGATCATGGTGCCtctAAATTGATAATTGCTAGCGTAAAATGC	Used for Orc4 mutagenesis
**YEH036**	GGTTGCCTTAAGGGCGgccGACGGATCTTTTAATTTTAATTTAGC	Used for Orc4 mutagenesis
**YEH037**	GCTAAATTAAAATTAAAAGATCCGTCggcCGCCCTTAAGGCAACC	Used for Orc4 mutagenesis
**YEH038**	GGTTGCCTTAAGGGCGgagGACGGATCTTTTAATTTTAATTTAGC	Used for Orc4 mutagenesis
**YEH039**	GCTAAATTAAAATTAAAAGATCCGTCctcCGCCCTTAAGGCAACC	Used for Orc4 mutagenesis
**YEH040**	CGTTGGTTTGAGAGATAATGCGgcgGCAGCATTTTACGCTAGC	Used for Orc4 mutagenesis
**YEH041**	GCTAGCGTAAAATGCTGCcgcCGCATTATCTCTCAAACCAACG	Used for Orc4 mutagenesis
**YEH042**	CGTTGGTTTGAGAGATAATGCGcggGCAGCATTTTACGCTAGC	Used for Orc4 mutagenesis
**YEH043**	GCTAGCGTAAAATGCTGCccgCGCATTATCTCTCAAACCAACG	Used for Orc4 mutagenesis

**YEH180**	CAGCAgctgccGCTAGCAATTATCAATTTCAG	Used for Orc4 mutagenesi s
**YEH181**	CTGAAATTGATAATTGCTAGCggcagcTGCTG	Used for Orc4 mutagenesi s
**YEH182**	CGACCGCAGCAattcaaGCTAGCAATTATCAATTTCAG	Used for Orc4 mutagenesi s
**YEH183**	CTGAAATTGATAATTGCTAGCttgaatTGCTGCGGTCG	Used for Orc4 mutagenesi s
**YEH184**	CGACCGCAGCAtactttGCTAGCAATTATCAATTTCAG	Used for Orc4 mutagenesi s
**YEH185**	CTGAAATTGATAATTGCTAGCaaagtaTGCTGCGGTCG	Used for Orc4 mutagenesi s
**YEH186**	CCGAGAAATCAGCCGTTGGTTTGaaaGATAATGCGAC	Used for Orc4 mutagenesi s
**YEH187**	GTCGCATTATCtttCAAACCAACGGCTGATTTCTCGG	Used for Orc4 mutagenesi s
**YEH188**	CGTTGGTTTGAGAGATAATGCGattGCAGCATTTTACGCTAG C	Used for Orc4 mutagenesi s
**YEH189**	GCTAGCGTAAAATGCTGCaatCGCATTATCTCTCAAACCAAC G	Used for Orc4 mutagenesi s
**YEH190**	CGTTGGTTTGAGAGATAATGCGgatGCAGCATTTTACGCTAG C	Used for Orc4 mutagenesi s
**YEH191**	GCTAGCGTAAAATGCTGCatcCGCATTATCTCTCAAACCAAC G	Used for Orc4

		mutagenesis
**YEH235**	ACATTTGCTGGGCCTgccAGGCAAACTTTATTATACAATC	Used for Orc4 mutagenesis
**YEH236**	GATTGTATAATAAAGTTTGCCTggcAGGCCCAGCAAATGT	Used for Orc4 mutagenesis
**YEH237**	ACATTTGCTGGGCCTcgcAGGCAAACTTTATTATACAATC	Used for Orc4 mutagenesis
**YEH238**	GATTGTATAATAAAGTTTGCCTgcgAGGCCCAGCAAATGT	Used for Orc4 mutagenesis
**YEH239**	ACATTTGCTGGGCCTGTGgccCAAACTTTATTATACAATC	Used for Orc4 mutagenesis
**YEH240**	GATTGTATAATAAAGTTTGggcCACAGGCCCAGCAAATGT	Used for Orc4 mutagenesis
**YEH241**	ACATTTGCTGGGCCTGTGgacCAAACTTTATTATACAATC	Used for Orc4 mutagenesis
**YEH242**	GATTGTATAATAAAGTTTGgtcCACAGGCCCAGCAAATGT	Used for Orc4 mutagenesis
**YEH304**	CCGAGAAATCAGCCGTTGGTTTGAGAagaAATGCGAC	Used for Orc4 mutagenesis
**YEH305**	GTCGCATTtctTCTCAAACCAACGGCTGATTTCTCGG	Used for Orc4 mutagenesis
**YEH352**	CCGCAGCATTTTACGCTAGCAATttgCAATTTCAGGGCACC	Used for Orc4 mutagenesis

**YEH353**	GGTGCCCTGAAATTGcaaATTGCTAGCGTAAAATGCTGCGG	Used for Orc4 mutagenesis
**YEH354**	CCGCAGCATTTTACGCTAGCAATagaCAATTTCAGGGCACC	Used for Orc4 mutagenesis
**YEH355**	GGTGCCCTGAAATTGtctATTGCTAGCGTAAAATGCTGCGG	Used for Orc4 mutagenesis
**YEH356**	CGCTAGCAATTATCAActaCAGGGCACCATGATCCCG	Used for Orc4 mutagenesis
**YEH357**	CGGGATCATGGTGCCCTGtagTTGATAATTGCTAGCG	Used for Orc4 mutagenesis
**YEH358**	CGCTAGCAATTATCAAtcaCAGGGCACCATGATCCCG	Used for Orc4 mutagenesis
**YEH359**	CGGGATCATGGTGCCCTGtgaTTGATAATTGCTAGCG	Used for Orc4 mutagenesis
**YEH192**	GACAAGAAGCGAAgccAAGTACTGGGGCAATCATGTG	Used for Orc2 mutagenesis
**YEH193**	CACATGATTGCCCCAGTACTTggcTTCGCTTCTTGTC	Used for Orc2 mutagenesis
**YEH194**	GACAAGAAGCGAAACTgctTACTGGGGCAATCATGTG	Used for Orc2 mutagenesis
**YEH195**	CACATGATTGCCCCAGTAagcAGTTTCGCTTCTTGTC	Used for Orc2 mutagenesis
**YEH196**	GACAAGAAGCGAAACTAAGgctTGGGGCAATCATGTG	Used for Orc2

		mutagenesis
**YEH197**	CACATGATTGCCCCAagcCTTAGTTTCGCTTCTTGTC	Used for Orc2 mutagenesis
**YEH198**	GACAAGAAGCGAAACTAAGTACgctGGCAATCATGTG	Used for Orc2 mutagenesis
**YEH199**	CACATGATTGCCagcGTACTTAGTTTCGCTTCTTGTC	Used for Orc2 mutagenesis
**YEH200**	ACTAAGTACTGGGGCgctCATGTGATTTTGCAGA	Used for Orc2 mutagenesis
**YEH201**	TCTGCAAAATCACATGagcGCCCCAGTACTTAGT	Used for Orc2 mutagenesis
**YEH326**	CTGAGTTGACAAGAAGCGAACATGTGATTTTGCAGATCCA	Used for Orc2 mutagenesis
**YEH327**	TGGATCTGCAAAATCACATGTTCGCTTCTTGTCAACTCAG	Used for Orc2 mutagenesis
**YEH328**	TGGTCCCCGCTGAGTTGACACATGTGATTTTGCAGATCCA	Used for Orc2 mutagenesis
**YEH329**	TGGATCTGCAAAATCACATGTGTCAACTCAGCGGGGACCA	Used for Orc2 mutagenesis
**YEH019**	ATGACTATAAGCGAAGCTCG	Used for Orc4 sequencing

**YEH020**	CTCAATTGGAACAGCAGTTG	Used for Orc4 sequencing /integrated NTAP-Orc4 sequencing
**YEH021**	AAGAGTGATTTATATGCCGC	Used for Orc4 sequencing /integrated NTAP-Orc4 sequencing
**YEH022**	TCAACTCCAGAATTCCCAC	Used for Orc4 sequencing /integrated NTAP-Orc4 sequencing
**YEH023**	TCACAGTTGTGTCCAGGAG	Used for Orc4 sequencing
**YEH221**	GGAAGACTTTGTAGAGCAT	Used for Orc2 sequencing
**YEH222**	CCTACACGTATGCGGAA	Used for Orc2 sequencing
**YEH223**	ATGCTCTACAAAGTCTTCC	Used for Orc2 sequencing
**YEH224**	TTCCGCATACGTGTAGG	Used for Orc2 sequencing
**gYEH003-F**	ATTTGTAATACGCTTTACTAGTTTT	Used for gRNA003 insertion to bRA89 plasmid

**gYEH003-R**	TAGTAAAGCGTATTACAAATGATCA	Used for gRNA003 insertion to bRA89 plasmid
**gYEH005-F**	GAATATACTAAAAAATGAGCGTTTT	Used for gRNA005 insertion to bRA89 plasmid
**gYEH005-R**	GCTCATTTTTTAGTATATTCGATCA	Used for gRNA005 insertion to bRA89 plasmid
**YEH263**	AGAAGAAGCATGGCAGGCCTTG	Used for CRISPR NTAP-Orc4 template construction/integrated NTAP-Orc4 sequencing
**YEH264**	accagcaccTAGGGCGAATTG	Used for CRISPR NTAP-Orc4 template construction
**YEH265**	CTAggtgctggtATGACTATAAG	Used for CRISPR NTAP-Orc4 template construction

**YEH266**	TTTTTCAACGCTAACTATAATTT	Used for CRISPR NTAP-Orc4 template constructio n/integrate d NTAP-Orc4 sequencing
**YEH267**	CTCGAGGCCACCAAGAAGAGAAAGAGAAGAGC	Used for CRISPR NTAP-Orc4 template constructio n
**YEH268**	AGGCCTGCCATGCTTCTTCTTTTGAATATTACCGATATT	Used for CRISPR NTAP-Orc4 template constructio n
**YEH311**	TTCCTGCAGCCCGGGGGATCGAAGAGAAAGAGAAGAGCCA	Used for CRISPR NTAP-Orc4 template constructio n
**YEH312**	GCTCCACCGCGGTGGCGGCCTTTTTCAACGCTAACTATAA	Used for CRISPR NTAP-Orc4 template constructio n

**ssYEH001**	AATGAGCAGGCAAGATAAACGAAGGCAAAGATGACAGAGCAGAAAGCCCGAAGAGAAAGAGAAGAGCCAGATATTGACTG	Used for CRIPSR NTAP-Orc4 integration yeast transformation (coupled with gRNA003)
**ssYEH002**	ACAAGTGGCAATGAAAAAAATTATAGTTAGCGTTGAAAAATgcgAAAGCGTATTACAAATGAAACCAAGATTCAGATTGC	Used for CRIPSR NTAP-Orc4 integration yeast transformation (coupled with gRNA003)
**ssYEH003**	TTATACATTATATAAAGTAATGTGATTTCTTCGAAGAATATACTAAAAAGAAGAGAAAGAGAAGAGCCAGATATTGACTG	Used for CRIPSR NTAP-Orc4 integration yeast transformation (coupled with gRNA005)
**ssYEH004**	ACAAGTGGCAATGAAAAAAATTATAGTTAGCGTTGAAAAAATatcCAGGCAAGATAAACGAAGGCAAAGATGACAGAGCA	Used for CRIPSR NTAP-Orc4 integration yeast transformation (coupled with gRNA005)

**YEH298**	TTAAGAGCTTGGTGAGCGCT	Used for CRISPR NTAP-Orc4 insertion checking
**YEH299**	ATGCTTGGCAGAGCATGTAT	Used for CRISPR NTAP-Orc4 insertion checking
**YEH173**	GTGCCCCGGAGGATGAGA	Used for integrated NTAP-Orc4 sequencing
**YEH279**	TCACCATCTTCAACAGTCAA	Used for integrated NTAP-Orc4 sequencing
**YEH281**	CGCGAATTGTGATATACCTA	Used for integrated NTAP-Orc4 sequencing
**YEH282**	ATATGAGTGATAATGGTTCC	Used for integrated NTAP-Orc4 sequencing
**YEH294**	GAAGAGAAAGAGAAGAGCCA	Used for integrated NTAP-Orc4 sequencing
**ARS416 L1 AR**	CTCGGCATTCCTGCTGAACCGCTCTTCCGATCT CAAATGATTTAGCATTATCTTTAC	Used for MPOS assay ARS416 sequencing library barcoding

**ARS317 L1 AR**	CTCGGCATTCCTGCTGAACCGCTCTTCCGATCT TTTTATGGAAGATTAAGCTCA	Used for MPOS assay ARS317 sequencing library barcoding
**ARS416 L1 AF01**	ACTCTTTCCCTACACGACGCTCTTCCGATCT ACAT AGACAAATGGTGTAAAAGAC	Used for MPOS assay ARS416 sequencing library barcoding
**ARS416 L1 AF02**	ACTCTTTCCCTACACGACGCTCTTCCGATCT AGCC AGACAAATGGTGTAAAAGAC	Used for MPOS assay ARS416 sequencing library barcoding
**ARS416 L1 AF03**	ACTCTTTCCCTACACGACGCTCTTCCGATCT ATGT AGACAAATGGTGTAAAAGAC	Used for MPOS assay ARS416 sequencing library barcoding
**ARS416 L1 AF04**	ACTCTTTCCCTACACGACGCTCTTCCGATCT CATA AGACAAATGGTGTAAAAGAC	Used for MPOS assay ARS416 sequencing library barcoding
**ARS416 L1 AF05**	ACTCTTTCCCTACACGACGCTCTTCCGATCT CGAT AGACAAATGGTGTAAAAGAC	Used for MPOS assay ARS416 sequencing library barcoding

**ARS416 L1 AF06**	ACTCTTTCCCTACACGACGCTCTTCCGATCT CTCT AGACAAATGGTGTAAAAGAC	Used for MPOS assay ARS416 sequencing library barcoding
**ARS416 L1 AF07**	ACTCTTTCCCTACACGACGCTCTTCCGATCT GAGA AGACAAATGGTGTAAAAGAC	Used for MPOS assay ARS416 sequencing library barcoding
**ARS416 L1 AF08**	ACTCTTTCCCTACACGACGCTCTTCCGATCT GCTA AGACAAATGGTGTAAAAGAC	Used for MPOS assay ARS416 sequencing library barcoding
**ARS416 L1 AF09**	ACTCTTTCCCTACACGACGCTCTTCCGATCT GTAT AGACAAATGGTGTAAAAGAC	Used for MPOS assay ARS416 sequencing library barcoding
**ARS416 L1 AF10**	ACTCTTTCCCTACACGACGCTCTTCCGATCT TACA AGACAAATGGTGTAAAAGAC	Used for MPOS assay ARS416 sequencing library barcoding
**ARS416 L1 AF11**	ACTCTTTCCCTACACGACGCTCTTCCGATCT TCGG AGACAAATGGTGTAAAAGAC	Used for MPOS assay ARS416 sequencing library barcoding

**ARS416 L1 AF12**	ACTCTTTCCCTACACGACGCTCTTCCGATCT TGTA AGACAAATGGTGTAAAAGAC	Used for MPOS assay ARS416 sequencing library barcoding
**ARS416 L1 AF13**	ACTCTTTCCCTACACGACGCTCTTCCGATCT AAAA AGACAAATGGTGTAAAAGAC	Used for MPOS assay ARS416 sequencing library barcoding
**ARS416 L1 AF14**	ACTCTTTCCCTACACGACGCTCTTCCGATCT ACCA AGACAAATGGTGTAAAAGAC	Used for MPOS assay ARS416 sequencing library barcoding
**ARS416 L1 AF15**	ACTCTTTCCCTACACGACGCTCTTCCGATCT AGGA AGACAAATGGTGTAAAAGAC	Used for MPOS assay ARS416 sequencing library barcoding
**ARS416 L1 AF16**	ACTCTTTCCCTACACGACGCTCTTCCGATCT ATTA AGACAAATGGTGTAAAAGAC	Used for MPOS assay ARS416 sequencing library barcoding
**ARS416 L1 AF17**	ACTCTTTCCCTACACGACGCTCTTCCGATCT CCAG AGACAAATGGTGTAAAAGAC	Used for MPOS assay ARS416 sequencing library barcoding

**ARS416 L1 AF18**	ACTCTTTCCCTACACGACGCTCTTCCGATCT CGCA AGACAAATGGTGTAAAAGAC	Used for MPOS assay ARS416 sequencing library barcoding
**ARS416 L1 AF19**	ACTCTTTCCCTACACGACGCTCTTCCGATCT CTGG AGACAAATGGTGTAAAAGAC	Used for MPOS assay ARS416 sequencing library barcoding
**ARS416 L1 AF20**	ACTCTTTCCCTACACGACGCTCTTCCGATCT GATT AGACAAATGGTGTAAAAGAC	Used for MPOS assay ARS416 sequencing library barcoding
**ARS416 L1 AF21**	ACTCTTTCCCTACACGACGCTCTTCCGATCT GGAA AGACAAATGGTGTAAAAGAC	Used for MPOS assay ARS416 sequencing library barcoding
**ARS416 L1 AF22**	ACTCTTTCCCTACACGACGCTCTTCCGATCT GTCA AGACAAATGGTGTAAAAGAC	Used for MPOS assay ARS416 sequencing library barcoding
**ARS416 L1 AF23**	ACTCTTTCCCTACACGACGCTCTTCCGATCT TAGC AGACAAATGGTGTAAAAGAC	Used for MPOS assay ARS416 sequencing library barcoding

**ARS416 L1 AF24**	ACTCTTTCCCTACACGACGCTCTTCCGATCT TCTC AGACAAATGGTGTAAAAGAC	Used for MPOS assay ARS416 sequencing library barcoding
**ARS317 L1 AF01**	ACTCTTTCCCTACACGACGCTCTTCCGATCT ACAT CAGTGTTTTCAATTTTTTATTAAAC	Used for MPOS assay ARS317 sequencing library barcoding
**ARS317 L1 AF02**	ACTCTTTCCCTACACGACGCTCTTCCGATCT AGCC CAGTGTTTTCAATTTTTTATTAAAC	Used for MPOS assay ARS317 sequencing library barcoding
**ARS317 L1 AF03**	ACTCTTTCCCTACACGACGCTCTTCCGATCT ATGT CAGTGTTTTCAATTTTTTATTAAAC	Used for MPOS assay ARS317 sequencing library barcoding
**ARS317 L1 AF04**	ACTCTTTCCCTACACGACGCTCTTCCGATCT CATA CAGTGTTTTCAATTTTTTATTAAAC	Used for MPOS assay ARS317 sequencing library barcoding
**ARS317 L1 AF05**	ACTCTTTCCCTACACGACGCTCTTCCGATCT CGAT CAGTGTTTTCAATTTTTTATTAAAC	Used for MPOS assay ARS317 sequencing library barcoding

**ARS317 L1 AF06**	ACTCTTTCCCTACACGACGCTCTTCCGATCT CTCT CAGTGTTTTCAATTTTTTATTAAAC	Used for MPOS assay ARS317 sequencing library barcoding
**ARS317 L1 AF07**	ACTCTTTCCCTACACGACGCTCTTCCGATCT GAGA CAGTGTTTTCAATTTTTTATTAAAC	Used for MPOS assay ARS317 sequencing library barcoding
**ARS317 L1 AF08**	ACTCTTTCCCTACACGACGCTCTTCCGATCT GCTA CAGTGTTTTCAATTTTTTATTAAAC	Used for MPOS assay ARS317 sequencing library barcoding
**ARS317 L1 AF09**	ACTCTTTCCCTACACGACGCTCTTCCGATCT GTAT CAGTGTTTTCAATTTTTTATTAAAC	Used for MPOS assay ARS317 sequencing library barcoding
**ARS317 L1 AF10**	ACTCTTTCCCTACACGACGCTCTTCCGATCT TACA CAGTGTTTTCAATTTTTTATTAAAC	Used for MPOS assay ARS317 sequencing library barcoding
**ARS317 L1 AF11**	ACTCTTTCCCTACACGACGCTCTTCCGATCT TCGG CAGTGTTTTCAATTTTTTATTAAAC	Used for MPOS assay ARS317 sequencing library barcoding

**ARS317 L1 AF12**	ACTCTTTCCCTACACGACGCTCTTCCGATCT TGTA CAGTGTTTTCAATTTTTTATTAAAC	Used for MPOS assay ARS317 sequencing library barcoding
**ARS317 L1 AF13**	ACTCTTTCCCTACACGACGCTCTTCCGATCT AAAA CAGTGTTTTCAATTTTTTATTAAAC	Used for MPOS assay ARS317 sequencing library barcoding
**ARS317 L1 AF14**	ACTCTTTCCCTACACGACGCTCTTCCGATCT ACCA CAGTGTTTTCAATTTTTTATTAAAC	Used for MPOS assay ARS317 sequencing library barcoding
**ARS317 L1 AF15**	ACTCTTTCCCTACACGACGCTCTTCCGATCT AGGA CAGTGTTTTCAATTTTTTATTAAAC	Used for MPOS assay ARS317 sequencing library barcoding
**ARS317 L1 AF16**	ACTCTTTCCCTACACGACGCTCTTCCGATCT ATTA CAGTGTTTTCAATTTTTTATTAAAC	Used for MPOS assay ARS317 sequencing library barcoding
**ARS317 L1 AF17**	ACTCTTTCCCTACACGACGCTCTTCCGATCT CCAG CAGTGTTTTCAATTTTTTATTAAAC	Used for MPOS assay ARS317 sequencing library barcoding

**ARS317 L1 AF18**	ACTCTTTCCCTACACGACGCTCTTCCGATCT CGCA CAGTGTTTTCAATTTTTTATTAAAC	Used for MPOS assay ARS317 sequencing library barcoding
**ARS317 L1 AF19**	ACTCTTTCCCTACACGACGCTCTTCCGATCT CTGG CAGTGTTTTCAATTTTTTATTAAAC	Used for MPOS assay ARS317 sequencing library barcoding
**ARS317 L1 AF20**	ACTCTTTCCCTACACGACGCTCTTCCGATCT GATT CAGTGTTTTCAATTTTTTATTAAAC	Used for MPOS assay ARS317 sequencing library barcoding
**ARS317 L1 AF21**	ACTCTTTCCCTACACGACGCTCTTCCGATCT GGAA CAGTGTTTTCAATTTTTTATTAAAC	Used for MPOS assay ARS317 sequencing library barcoding
**ARS317 L1 AF22**	ACTCTTTCCCTACACGACGCTCTTCCGATCT GTCA CAGTGTTTTCAATTTTTTATTAAAC	Used for MPOS assay ARS317 sequencing library barcoding
**ARS317 L1 AF23**	ACTCTTTCCCTACACGACGCTCTTCCGATCT TAGC CAGTGTTTTCAATTTTTTATTAAAC	Used for MPOS assay ARS317 sequencing library barcoding

**ARS317 L1 AF24**	ACTCTTTCCCTACACGACGCTCTTCCGATCT TCTC CAGTGTTTTCAATTTTTTATTAAAC	Used for MPOS assay ARS317 sequencing library barcoding
**YEH229**	GCCGCAAAAAAGGGAATAAG	Used for MPOS assay sequencing library contrusction
**YEH230**	CGTGTTCAAACGATACCTGG	Used for MPOS assay sequencing library contrusction
**YEH231**	AATGATACGGCGACCACCGAGATCTACACTCTTTCCCTACACG	Used for MPOS assay sequencing library contrusction
**YEH232**	AAGCAGAAGACGGCATACGAGATCGGTCTCGGCATTCCTGCT	Used for MPOS assay sequencing library contrusction

Supplementary Methods Table 2 | Primers and oligos sequences used in this study
