# Supplementary material for: Evolution of DNA replication origin specification and gene silencing mechanisms
Source: Nat Commun. 2020 Oct 14;11:5175. doi: 10.1038/s41467-020-18964-x (PMC7560902; doi:10.1038/s41467-020-18964-x)
Supplement: Supplementary file 3 — Description of Additional Supplementary Files [file 41467_2020_18964_MOESM3_ESM.pdf]

Description of Additional Supplementary Files

File Name: Supplementary Movie 1

Description: **Interaction of the ORC4 α-helix and origin DNA.** The Movie was made with PyMOL software (The PyMOL Molecular Graphics System, Version 2.0 Schrödinger, LLC). F485 and Y486 highlighted in red. R478 and N489 highlighted in orange. V475 and A487 highlighted in brown. The 5' to 3' origin DNA strand is in green color with A/T29, G/T30 logo positions highlighted in Dark purple, while the opposite strand is in grey color with T/A29', C/A30' logo positions highlighted in Light purple.
