# Supplementary material for: Evolution of DNA replication origin specification and gene silencing mechanisms
Source: Nat Commun. 2020 Oct 14;11:5175. doi: 10.1038/s41467-020-18964-x (PMC7560902; doi:10.1038/s41467-020-18964-x)
Supplement: Supplementary file 5 — Reporting Summary [file 41467_2020_18964_MOESM5_ESM.pdf]

nature research

Corresponding author(s): Bruce Stillman

Last updated by author(s): August 11, 2020

Reporting Summary

Nature Research wishes to improve the reproducibility of the work that we publish. This form provides structure for consistency and transparency in reporting. For further information on Nature Research policies, see our Editorial Policies and the Editorial Policy Checklist.

Statistics

For all statistical analyses, confirm that the following items are present in the figure legend, table legend, main text, or Methods section.

n/a	Confirmed	
☐	☒	The exact sample size (n) for each experimental group/condition, given as a discrete number and unit of measurement
☐	☒	A statement on whether measurements were taken from distinct samples or whether the same sample was measured repeatedly
☐	☒	The statistical test(s) used AND whether they are one- or two-sided<br>*Only common tests should be described solely by name; describe more complex techniques in the Methods section.*
☐	☒	A description of all covariates tested
☐	☒	A description of any assumptions or corrections, such as tests of normality and adjustment for multiple comparisons
☐	☒	A full description of the statistical parameters including central tendency (e.g. means) or other basic estimates (e.g. regression coefficient) AND variation (e.g. standard deviation) or associated estimates of uncertainty (e.g. confidence intervals)
☐	☒	For null hypothesis testing, the test statistic (e.g. F, t, r) with confidence intervals, effect sizes, degrees of freedom and P value noted<br>*Give P values as exact values whenever suitable.*
☒	☐	For Bayesian analysis, information on the choice of priors and Markov chain Monte Carlo settings
☒	☐	For hierarchical and complex designs, identification of the appropriate level for tests and full reporting of outcomes
☒	☐	Estimates of effect sizes (e.g. Cohen's d, Pearson's r), indicating how they were calculated

Our web collection on statistics for biologists contains articles on many of the points above.

Software and code

Policy information about availability of computer code

Data collection	N/A
Data analysis	Read mapping was performed using BWA and SAMtools. Other analyses were performed using Python scripts and Jupyter notebooks that are available at https://github.com/jbkinney/17_ars.

For manuscripts utilizing custom algorithms or software that are central to the research but not yet described in published literature, software must be made available to editors and reviewers. We strongly encourage code deposition in a community repository (e.g. GitHub). See the Nature Research guidelines for submitting code & software for further information.

Data

Policy information about availability of data

All manuscripts must include a data availability statement. This statement should provide the following information, where applicable:

- Accession codes, unique identifiers, or web links for publicly available datasets
- A list of figures that have associated raw data
- A description of any restrictions on data availability

Raw sequencing data has been deposited on the NCBI Sequence Read Archive under accession number PRJNA595459. No figures have associated raw data. All data reported in this publication is available at PRJNA595459, at https://github.com/jbkinney/17_ars, or from the authors upon request.

Field-specific reporting

Please select the one below that is the best fit for your research. If you are not sure, read the appropriate sections before making your selection.

☒ Life sciences ☐ Behavioural & social sciences ☐ Ecological, evolutionary & environmental sciences

For a reference copy of the document with all sections, see nature.com/documents/nr-reporting-summary-flat.pdf

Life sciences study design

All studies must disclose on these points even when the disclosure is negative.

Sample size	N/A
Data exclusions	See text.
Replication	See text.
Randomization	N/A
Blinding	N/A

Reporting for specific materials, systems and methods

We require information from authors about some types of materials, experimental systems and methods used in many studies. Here, indicate whether each material, system or method listed is relevant to your study. If you are not sure if a list item applies to your research, read the appropriate section before selecting a response.

Materials & experimental systems

n/a	Involved in the study	
☐	☒	Antibodies
☒	☐	Eukaryotic cell lines
☒	☐	Palaeontology and archaeology
☒	☐	Animals and other organisms
☒	☐	Human research participants
☒	☐	Clinical data
☒	☐	Dual use research of concern

Methods

n/a	Involved in the study	
☐	☒	ChIP-seq
☐	☒	Flow cytometry
☒	☐	MRI-based neuroimaging

Antibodies

Antibodies used	monoclonal antibodies directed agains two ORC subunits, made in the Stillman laboratory. Orc4 (SB12) used at 1:2000 dilution and Orc1 (SB13) used at 1:1000 dilution. Also IgG Sepharose 6 Fast Flow beads (GE Healthcare, Cat# 17-0969-01)
Validation	These antibodies have been used in many studies of yeast ORC since thy were made in the early 1990s.

ChIP-seq

Data deposition

☒ Confirm that both raw and final processed data have been deposited in a public database such as GEO.

☒ Confirm that you have deposited or provided access to graph files (e.g. BED files) for the called peaks.

Data access links<br>*May remain private before publication.*	Raw data is available on the Sequence Read Archive at PRJNA595459. Analysis scripts are available at https://github.com/jbkinney/17_ars. No peak calling was performed in this study, so there are no graph files to provide.
Files in database submission	All files at PRJNA595459. and https://github.com/jbkinney/17_ars.
Genome browser session<br>(e.g. UCSC)	N/A

Methodology

Replicates	See text.
Sequencing depth	See text.
Antibodies	See text.
Peak calling parameters	N/A
Data quality	N/A
Software	BAM and SAMtools were used to do read mapping. No peak calling was performed. All ChIP-seq analysis was performed using custom Python scripts at https://github.com/jbkinney/17_ars.

Flow Cytometry

Plots

Confirm that:

- [ ] The axis labels state the marker and fluorochrome used (e.g. CD4-FITC).
- [ ] The axis scales are clearly visible. Include numbers along axes only for bottom left plot of group (a 'group' is an analysis of identical markers).
- [ ] All plots are contour plots with outliers or pseudocolor plots.
- [ ] A numerical value for number of cells or percentage (with statistics) is provided.

Methodology

Sample preparation	The above parameters are not relevant to the type of flow cytometry we used. We did not sort and did not perform two stain flow sorts. Flow cytometry was was used to determine the cell cycle distribution only and DNA was stained with SYBR green as stated in the figure legend.
Instrument	BD LSRFortessa Dual Special Order System instrument
Software	BD FACSDiva Software Version 8.0.1 Firmware Version 1.4 (BD LSRFortessa)
Cell population abundance	not applicable
Gating strategy	FlowJo Version 10.6.1 was used to analyze the data and no gating strategy used.

- [ ] Tick this box to confirm that a figure exemplifying the gating strategy is provided in the Supplementary Information.
