# Supplementary material for: Evolution of DNA replication origin specification and gene silencing mechanisms
Source: Nat Commun. 2020 Oct 14;11:5175. doi: 10.1038/s41467-020-18964-x (PMC7560902; doi:10.1038/s41467-020-18964-x)

Supplementary Fig. 4 anti-Orc4 (short exposure)

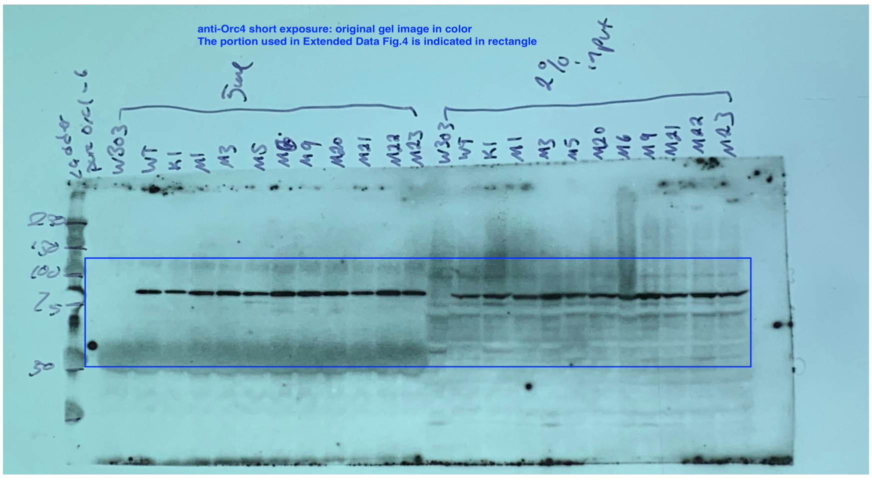

anti-Orc4 (long exposure)

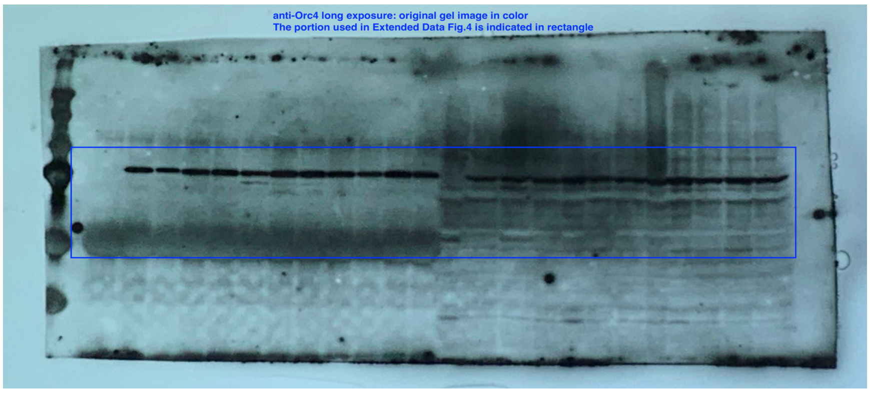

anti-Orc1 (short exposure)

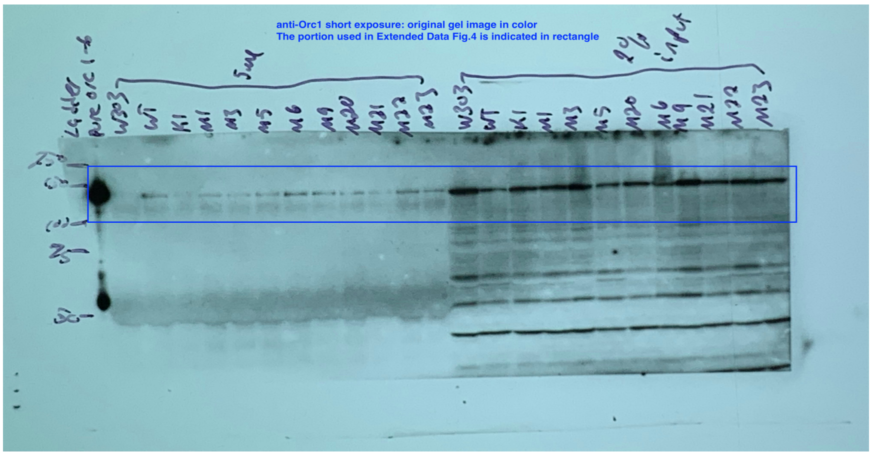

anti-Orc1 (long exposure)

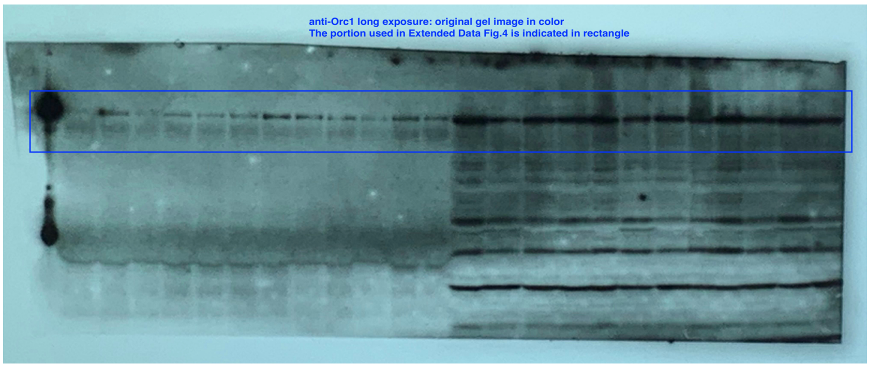

Supplement: Supplementary file 6 — Source Data [file 41467_2020_18964_MOESM6_ESM.pdf]
